# Older adult care policy implications derived from quality of life disparities and sociodemographic risk factors

**DOI:** 10.3389/fpubh.2026.1907290

**Published:** 2026-10-09

**Authors:** Yulin Tu, Chao He, Tangbiyao Tan, Yanhong Zhou, Huakang Zhu

**Affiliations:** 1 Hunan Normal University, Changsha, China; 2 Yonsei University, Seoul, Republic of Korea; 3 Hunan University of Finance and Economics, Changsha, China

**Keywords:** aging population, older adults care policy, public health, quality of life, social inequality, sociodemographic disparities, sustainable development, world development indicators

## Abstract

**Inroduction:**

Population aging is placing increasing pressure on healthcare and social welfare systems worldwide, yet empirical evidence on whether integrated care policies for older adults reduce quality-of-life inequalities and improve well-being remains limited. This study examines the effects of such policies, together with socioeconomic, healthcare-access, and demographic factors, on the quality of life of older adults across countries.

**Methods:**

A balanced panel of 24 countries observed annually from 2018 to 2025 (N = 192 country-years) was assembled from World Bank (World Development Indicators, Policy Database, ASPIRE) and WHO Global Health Observatory data. Eleven countries that adopted a national integrated older adult care policy in 2021–2022 formed the treatment group, and 13 non-adopting countries formed the control group. The dependent variables were a multidimensional Quality of Life Index (QoL; health, economic security, and social participation on a 0–100 scale) and life expectancy at age 60 (LE60). A difference-in-differences (DID) framework with country and year fixed effects was complemented by event-study analysis, mechanism tests, robustness checks, propensity score matching DID (PSM-DID), and heterogeneity analysis.

**Results:**

Policy adoption was associated with a 3.842-point increase in QoL (95% CI: 2.39–5.30; *p* < 0.01), or 0.33 standard deviations, and a 1.126-year increase in LE60 (95% CI: 0.50–1.75; *p* < 0.01; 0.36 SD). Pre-policy event-study coefficients were individually and jointly insignificant (joint *p* = 0.91), consistent with parallel trends, whereas post-policy effects rose from 1.264 (95% CI: 0.25–2.28) in the adoption year to 3.842 (95% CI: 2.39–5.30) three years later. The policy increased universal health coverage (*β* = 4.126) and social protection coverage (*β* = 5.284) and reduced out-of-pocket health expenditure (*β* = −3.417). Results were robust to an alternative outcome, a lagged specification, and PSM-DID; the point estimate was larger in lower-income countries, although the subsample confidence intervals overlapped.

**Discussion:**

Under the identifying assumptions of the DID design, integrated care policies for older adults substantially improve quality of life and life expectancy at age 60, operating mainly through expanded health coverage, broader social protection, and a lower financial burden, and may help narrow welfare inequalities among older adults.

## 1 Introduction

A multifaceted measure of older adults well-being, which can be interpreted through the interplay of physical, mental, social, and environmental parameters, health-related quality of life (HRQoL) has gained considerable significance with the worldwide aging population and rising healthcare concerns (1). Rapid population aging causes issues for health care and social support as well as older adults well-being; HRQoL and its policy concerns, therefore, gain increasing significance in the context of a rapidly growing older population (2). In the wake of escalating rates of population aging, HRQoL has received considerable significance, thereby pointing to the need for community-based healthcare, social support, and various public health policies that address issues concerning independent living and well-being of older adults (3). Rapid population aging and increased life expectancy lead to intensified concerns regarding older adults well-being, highlighting the importance of quality of life, healthcare accessibility, and socio-economic support through public health policies that address age-related issues (4). Reduction in HRQoL significantly occurs with hypertension, which points to the need for healthcare interventions and public policies aimed at better physical, mental, and social well-being (5). Sociodemographic, nutritional, environmental, and behavioral characteristics contribute to HRQoL, stressing the importance of precise public health policies in the improvement of population health and reduction of risk of cardiometabolic diseases (6). The growing aging population led to an increase in concern about disability and quality of life among older adults, which calls for adequate healthcare and aging public policies focused on maintaining independent living (7). Social disadvantage affects HRQoL, and health-related inequalities related to the impact on quality of life have been largely unknown, which is why equal access to healthcare, socioeconomic policy, and better social support should be emphasized (8). Physical health, mental health, social well-being, anxiety, and depression affect the quality of life of older adults, highlighting the importance of healthcare and aging policies in their amelioration (9). Work-related musculoskeletal disorders (WRMSDs) have been found to negatively impact the quality of life, physical health, and productivity of teachers, which therefore calls for work-related public health policies, ergonomic strategies, and welfare provisions in workplaces (10). Pain represents a substantial global health issue that impacts quality of life; however, the social and economic effects and relation to older adult well-being are scarce in the Indian population (11). Caregiver burden is escalating in sub-Saharan Africa, impacting caregivers’ health-related quality of life. There has been no research conducted on similar aspects of older adults in Ghana (12). Despite better survival rates, gastric cancer survivors endure the fear of recurrence and lower health-related quality of life; the factors that may affect fear of cancer recurrence (FCR) in Korean survivors have not been sufficiently studied (13). Individuals’ quality of life is significantly impacted by socioeconomic determinants of health; however, the cumulative effect of social disadvantage on quality of life, using a multidimensional framework and population-based data, is absent (14). Patients with oral cancer experience poor physical and psychological consequences as well as demoralization, which lowers their quality of life and raises their risk of suicidal thoughts. There has not been much discussion of the connection between oral cancer and patients’ spiritual requirements (15).

This research aims to assess how older adults care policies affect life expectancy and quality-of-life metrics in aging populations. It analyzes the mechanisms by which socioeconomic status, access to health care, demographic burden, and welfare provision impact the quality of life of older adults across countries. Using a difference-in-differences (DID) framework, the research explores policy effects, transmission mechanisms, and cross-country disparities to facilitate evidence-based policymaking for older adult care. The main key contributions are as follows:

Provides quasi-experimental evidence on the effectiveness of older adult care policies using a DID approach.
Simultaneously evaluates both a multidimensional Quality of Life Index (QoL) and Life Expectancy at Age 60 (LE60) as key indicators of the well-being of older adults.
Explains how older adult care policies improve outcomes through Universal Health Coverage (UHC), Social Protection Coverage (SPC), and reduced out-of-pocket health expenditure (OOP).
Identifies heterogeneous policy effects across countries, indicating stronger benefits in economically disadvantaged areas and providing guidance for targeted policy interventions.

Three terms are used consistently in this manuscript. Throughout the manuscript, “older adults” refers to persons aged 60 years and above, following the World Health Organization convention; “aging populations” refers to national populations undergoing demographic aging; and “older adult care policy” is retained as the established policy label for national programs targeting this age group. The abbreviations used in the analysis are defined at first appearance and summarized in Table 1.

**Table 1:** Key variables and measurements for examining older adult care policy implications, quality-of-life disparities, and sociodemographic risk factors.

Type	Variable	Symbol	Description	Source
DV	Quality of life index	QoL	Composite 0–100 index of older adults’ well-being covering health, economic security, and social participation (Section 3.2.1)	Authors’ construction from WDI, ASPIRE, and WHO indicators
	Life expectancy at age 60	LE60	Expected remaining lifespan of the population aged 60+	WDI
IV	Older adult care policy	POLICY	Treatment-group dummy (1 = country adopted a qualifying national older adult care policy in 2021–2022; 0 = non-adopting country)	World Bank Policy Database
	DID interaction term	DID	POLICY × POST, where POST = 1 from the adoption year onward, captures the policy effect	Authors’ construction
	GDP per capita	GDPPC	Economic development level (constant USD)	WDI
	Gini index	GINI	Income inequality measure	WDI
	Universal health coverage index	UHC	Coverage of essential health services	WHO / WDI
	Old-age dependency ratio	EDR	Ratio of the population aged 65+ to the working-age population	WDI
Control	Poverty rate	POV	Share of population below the poverty line	WDI
	Education level	EDU	Average years of schooling (human capital proxy)	WDI
	Health expenditure	HEXP	Health spending as % of GDP	WDI
	Out-of-pocket spending	OOP	Out-of-pocket health expenditure as % of current health expenditure	WDI
	Social protection coverage	SPC	Coverage of social welfare programs	World Bank ASPIRE
	Employment rate of older adults	EEMP	Labor participation rate of the older population	WDI

The remaining parts of this research are structured as follows. Section 2 is a literature review on the quality of life of older adults, an aging population, older adult care policies, and socio-demographic factors of risk. Section 3 is a description of research design, including data collection, measurement of variables, and statistical tests. Section 4 presents the empirical analysis, which includes descriptive statistics, a balance test, baseline DID results, a parallel trends test, event study analysis, tests of mechanisms, robustness tests, the estimation of PSM-DID, and heterogeneous analysis, among other components. Section 5 discusses the findings, and Section 6 presents the conclusion, policy implications, limitations, and suggestions for future research.

## 2 Related work

The existing methods used for the welfare assessment of older adults cannot effectively measure multidimensional QoL, address non-linear sociodemographic disparities, or detect the impact of older adult care policies on well-being. Table 2 outlines the existing literature on QoL in older adults and sociodemographic disparities.

**Table 2:** Summary of empirical literature on older adults quality of life, sociodemographic disparities, and health outcomes.

Ref no.	Objective	Methods	Key Findings	Limitations
Rico-Blázquez et al. (20)	To analyze gender-based factors influencing health-related quality of life among older informal caregivers in primary healthcare settings.	Observational cross-sectional multicenter study using EQ-5D and robust linear regression analysis.	Depression and caregiving burden significantly reduced caregivers’ quality of life, especially among women caregivers.	Cross-sectional design limits causal inference and long-term outcome evaluation.
Yao et al. (21)	To estimate and decompose income-related inequality in health-related quality of life among adults across mainland China populations.	Cross-sectional nationwide survey using EQ-5D-5L, concentration index, and Wagstaff decomposition analysis.	Income inequality, chronic conditions, and health literacy significantly influenced HRQoL disparities across Chinese populations.	A cross-sectional design restricts causal interpretation and temporal relationship analysis.
Ghanem et al. (22)	To investigate how lifestyle and sociodemographic factors affect oral health outcomes in the country’s representative adult population in Hungary.	Cross-sectional EHIS survey using Chi-square tests and multivariate logistic regression analyses.	Education, income, smoking, and regular dental visits significantly influenced oral health outcomes and perceptions.	Cross-sectional design limits causal inference and longitudinal health outcome assessment.
Alliu et al. (23)	To use longitudinal data to assess the humanistic and economic costs of multimorbidity among individuals in the United States.	Weighted estimation and generalized estimating equation (GEE) models with AR correlation are used in the MEPS longitudinal analysis.	Multimorbidity increased healthcare expenditures and reduced health-related quality of life consistently over time among adults.	Self-reported survey data may introduce reporting bias and measurement inaccuracies.
Liu et al.(24)	To investigate how digital health literacy affects older adults’ HRQoL and how a healthy lifestyle acts as a mediator.	Cross-sectional survey using Pearson correlation and SPSS PROCESS mediation analysis among community-dwelling older adults.	Digital health literacy significantly improved HRQoL through healthier lifestyle behaviors among older adults.	Cross-sectional design limits causal inference and temporal relationship evaluation.
Sieber et al. (25)	To examine how multimorbidity affects quality of life through functional and emotional mediators, and whether sociodemographic factors modify the differences in pathways.	Data from SHARE Waves 4–8 (*n* = 36,908). Longitudinal causal mediation and moderated mediation analyses assessed ADL, IADL, loneliness, and depressive symptoms.	Multimorbidity reduced quality of life; ADL, IADL, depression mediated effects; loneliness showed no mediation pathways.	Observational design, self-reported measures, residual confounding, limited generalizability outside Europe
Ramalivhana et al. (26)	To examine functional performance, health-related quality of life, and risk factors for non-communicable diseases between low-income urban and rural populations	Cross-sectional study of 311 adults using objective physical, biochemical, functional, and HRQoL measurements with statistical group comparisons	Urban participants showed higher NCD risk and poorer function; rural participants had better HRQoL outcomes.	Cross-sectional design, limited sample size, self-selection, regional restriction, and confounding factors
Prati (27)	To identify psychosocial correlates and predict quality of life, happiness, and life satisfaction in older European adults using machine learning	SHARE Wave 7 data (62,500 participants). Machine learning regression models analyzed psychosocial, health, and sociodemographic predictors.	Models explained variance in QoL, life satisfaction, happiness, health, and subjective factors as the most influential predictors.	Cross-sectional data, self-report bias, model interpretability limitations, and unmeasured confounders
Nagy et al. (28)	To assess the prevalence and risk factors of anxiety and depression and their association with quality of life in hemodialysis patients	Cross-sectional study of 298 HD patients using HADS for anxiety/depression and KDQOL-36 for quality-of-life assessment	High anxiety and depression prevalence are associated with inactivity, unemployment, longer dialysis duration, and poorer quality of life.	Cross-sectional design, self-report bias, single-country sample, limited causal inference
Geprägs et al. (29)	To assess changes in quality of life and pinpoint risk factors for lower life satisfaction in Germany during the COVID-19 epidemic.	Population-based cross-sectional survey of 2,515 German adults analyzing sociodemographic, mental, and physical health factors	Most reported stable QoL; mental health, income, age, and living situation influenced outcomes significantly	Cross-sectional design, self-report bias, recall bias, and limited causal inference
Muneera et al. (30)	To determine the prevalence of intrinsic capacity and investigate lifestyle and sociodemographic factors associated with intrinsic ability in older Indian individuals.	LASI Wave 1 (2017–18), 24,136 adults aged ≥60. Descriptive, bivariate, multivariable linear and logistic regression analyses performed	Higher IC linked to physical activity and education; tobacco, alcohol, and aging reduced capacity significantly	Cross-sectional design, self-reported data, recall bias, unmeasured confounding
Mihevc et al. (31)	To assess health-related quality of life and identify risk factors for low HRQoL among older adults with type 2 diabetes	Multicenter cross-sectional survey of 358 older adults with T2D using EQ-5D, ADS, and logistic regression analyses	Low HRQoL associated with higher BMI, depression, older age, and poorer diabetes appraisal scores	Cross-sectional design, self-reported measures, limited generalizability, potential selection bias
Liang et al. (32)	To investigate the relationship between health-related quality of life, multimorbidity, and chronic illnesses in older Chinese community-dwelling adults.	Cross-sectional study of 1,218 adults in three Chinese cities using EQ-5D-3L, regression, Tobit, and generalized linear models	Multimorbidity and chronic diseases significantly reduced HRQoL; stroke and COPD showed the strongest negative effects.	Cross-sectional design, self-report bias, limited geographic coverage, causal inference not possible
Ko and Lee (33)	To investigate the relationship between health-related quality of life and social frailty in older South Korean adults and to pinpoint contributing elements.	Cross-sectional secondary analysis of 735 older adults using EQ-5D, social frailty scale, and Tobit regression analysis	Social frailty is significantly associated with lower HRQoL; a high prevalence of frailty and pre-frailty is observed.	Cross-sectional design, self-reported data, limited generalizability, secondary data analysis
Bally et al. (34)	To identify factors associated with physical and mental health-related quality of life among community-dwelling older adults, longitudinally	Longitudinal study of 661 older adults using SF-12 surveys, baseline and follow-up, analyzed with linear regression models	Frailty, ADL limitations, multimorbidity, loneliness reduced HRQoL; some unexpected positive associations with age and living alone	Observational design, self-report bias, limited sample, residual confounding possible
Wang et al. (35)	To examine trends in quality of life among the Chinese population from 2008 to 2020 and differences across socio-demographic groups	Repeated cross-sectional Health Services Surveys (2008, 2013, 2020), EQ-5D mapping, utility conversion, and multiple linear regression analyses	QoL declined over time; worse reductions were observed among women, older adults, the unemployed, and the disadvantaged groups.	Cross-sectional waves, measurement mapping errors, regional limitation, and self-report bias

ADL = activities of daily living; ADS = Appraisal of Diabetes Scale; AR = autoregressive; IADL = instrumental activities of daily living; BMI = body mass index; COPD = chronic obstructive pulmonary disease; EHIS = European Health Interview Survey; EQ-5D = EuroQol five-dimension instrument; GEE = generalized estimating equation; HADS = Hospital Anxiety and Depression Scale; HD = hemodialysis; HRQoL = health-related quality of life; IC = intrinsic capacity; KDQOL-36 = Kidney Disease Quality of Life instrument; LASI = Longitudinal Ageing Study in India; MEPS = Medical Expenditure Panel Survey; NCD = non-communicable disease; SF-12 = 12-item Short Form Health Survey; SHARE = Survey of Health, Ageing and Retirement in Europe; T2D = type 2 diabetes.

### 2.1 Research gap

Most of the current cross-sectional and self-reported studies suffer from the inability to make causal inferences and lack measurements of longitudinal impact, as well as the overall efficacy of older adult care policy on their quality of life. The Hungarian study related to oral health presented in Table 2 uses a cross-sectional design; hence, no causal inferences can be made, while the use of self-reported measures increases the risk of recall bias and confounding variables. Similarly, the study about digital health literacy in Chongqing is characterized by the same design issue, self-reported measures, and is limited to only one geographical area of one country. As a result, there is a risk of residual confounding, and the results are not generalizable. The machine learning study on older Europeans uses cross-sectional data, hard-to-interpret models, and involves risks of self-report bias and residual confounding because of the high number of predictor variables. Contrary to longitudinal studies, most cross-sectional and self-reported studies fail to prove causality.

Such issues will be resolved using the proposed research, which involves longitudinal panel data, a DID approach, and a PSM-DID method to decrease selection bias and increase comparability of treatment and control groups, as well as multidimensional old-age welfare indices. This study is expected to support stronger inferences about policy effects under the DID assumptions, use fewer self-reported measures, increase cross-country comparability, and properly assess the impact of older adults care policies in various social and economic circumstances.

### 2.2 Hypothesis development

The hypotheses focus on the impact of older adult care policy and socioeconomic factors on quality of life and life expectancy, with reference to such factors as GDP, economic inequality, accessibility of health services, and pressure from demographic aging. The conceptual framework of the hypotheses is provided in Figure 1.

**Figure 1:**
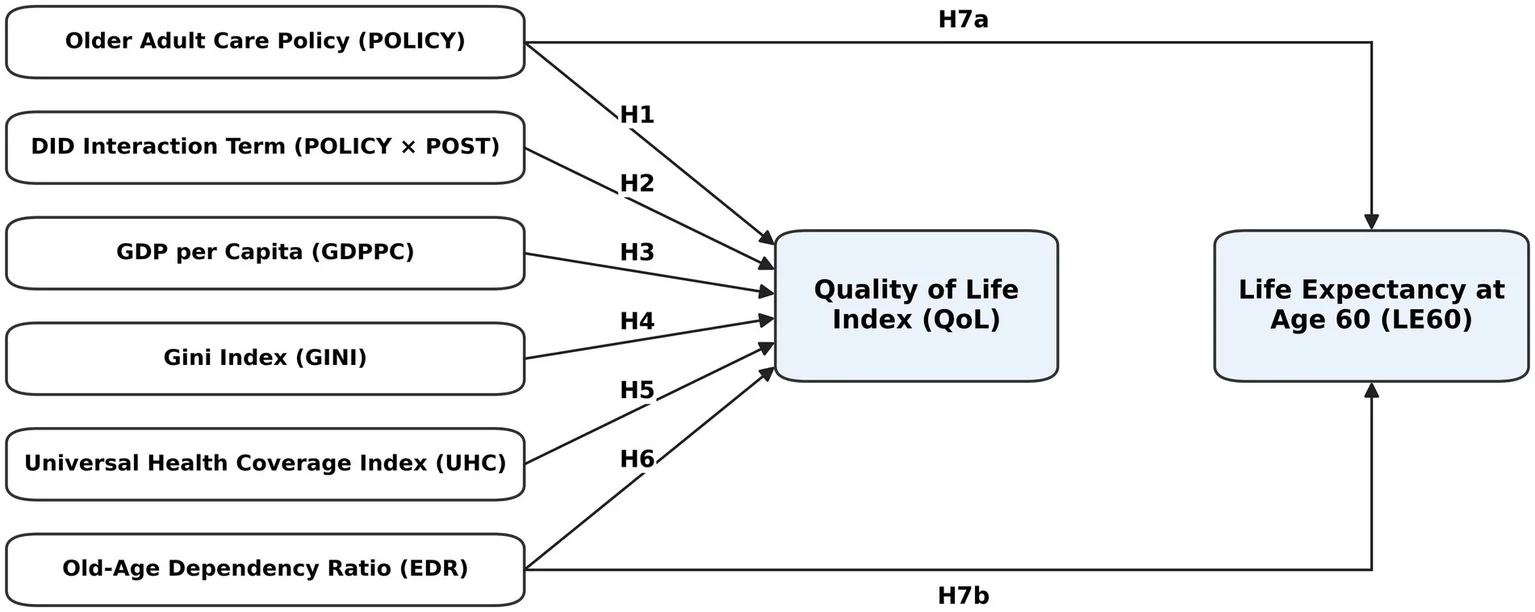
Conceptual framework linking older adult care policy and socioeconomic factors.

#### 2.2.1 H1: Older adult care policy (POLICY) → Quality of life index (QoL)

Older adult care policy implementation leads to a higher Quality-of-Life Index (QoL). The implementation of older adult care policies is predicted to achieve better healthcare, social welfare, and community services in the adopting country. An improvement in health status, financial situation, and social inclusion resulting from better social welfare would enhance the life satisfaction of the older adult population.

#### 2.2.2 H2: DID interaction term (POLICY × POST) → Quality of life index (QoL)

The DID interaction effect significantly improves the QoL. The treatment in the time between policy implementation and the post-policy period captures the effect of the older adults care interventions under the parallel-trends assumption. The treated countries are expected to show a significant improvement in older adults welfare compared to the control countries.

#### 2.2.3 H3: GDP per capita (GDPPC) → Quality of life index (QoL)

Higher GDP per capita leads to a higher quality-of-life index (QoL). Economically advanced countries generally have superior healthcare services, higher incomes, and a better public service system, all contributing to an enhancement of the living environment and welfare status for older adults.

#### 2.2.4 H4: Gini index (GINI) → Quality of life index (QoL)

Higher income inequality leads to a lower Quality-of-Life Index (QoL). High income inequality also leads to poor access to healthcare, social protection, and economic resources within a country, affecting both the health and social inclusion of disadvantaged older adults.

#### 2.2.5 H5: Universal health coverage index (UHC) → Quality of life index (QoL)

Higher universal health coverage leads to a higher Quality-of-Life Index (QoL). Enhanced access to needed healthcare services allows older adults to have appropriate medical care, preventive services, and continuous health management, leading to a greater sense of satisfaction.

#### 2.2.6 H6: Old-age dependency ratio (EDR) → Quality of life index (QoL)

A higher old-age dependency ratio leads to a lower Quality-of-Life Index (QoL). As the older adults’ dependency ratio goes up, so does the strain on pension systems, healthcare services, and the working population. Such increased old-age dependency may also decrease the resources available and the services offered to older adults, thus compromising their welfare.

#### 2.2.7 H7a: Older adult care policy (POLICY) → Life expectancy at age 60 (LE60)

Older adult care policy implementation leads to higher life expectancy at age 60. Good older adult care policies enhance the access of older adults to health care services, prevent and control diseases, and support the health and well-being of older adults by increasing survival rates and maintaining a healthy lifestyle.

#### 2.2.8 H7b: Old-age dependency ratio (EDR) → Life expectancy at age 60 (LE60)

A higher old-age dependency ratio negatively affects life expectancy at age 60. High proportions of older dependents in a country might lead to limitations in health and social welfare resources and thus may weaken older adult care provisions and possibly decrease life expectancy.

## 3 Methodology

To determine the link between the influence of older adult care policies on the quality-of-life gap and sociodemographic risk factors among older adults, a balanced panel of 24 countries was established, which was observed for a period of 8 years (from 2018 to 2025) using data from the WDI and World Bank Policy Databases. This dataset covers indicators related to the well-being of older adults, access to health services, socioeconomic and demographic conditions, and policy execution at the country level. Using Difference-in-Differences (DID) for estimation, the effect of older adult care policy on QoL and LE60 is estimated. The country fixed effects capture any difference that may exist across countries and do not vary over time (such as the nature of healthcare systems and economic development levels). The year fixed effects account for exogenous events such as the COVID-19 pandemic, while time-varying control variables deal with the remainder of heterogeneities. The parallel trend assumption was checked using an event study test. Mechanism and robustness tests are also conducted, and PSM-DID and heterogeneity tests are used to address selection bias. Figure 2 depicts the entire process flowchart.

**Figure 2:**
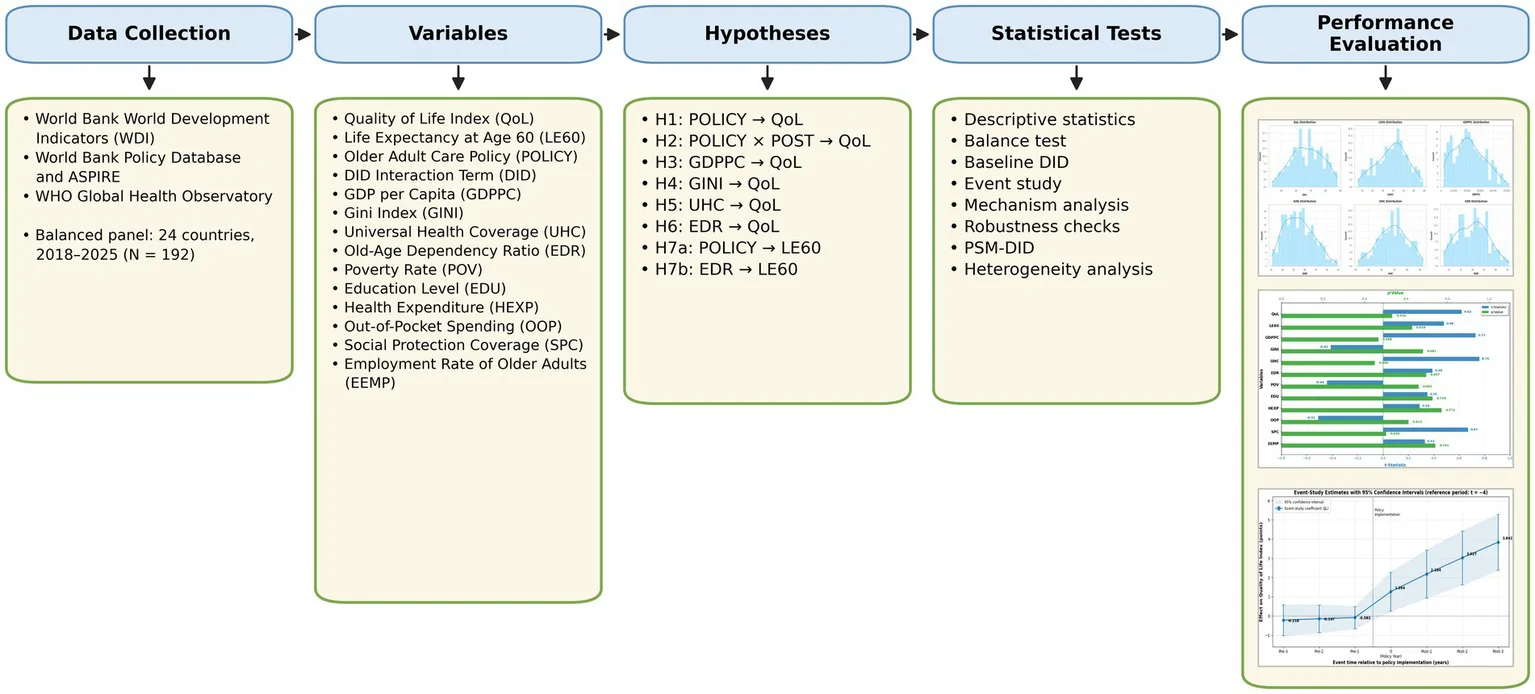
Methodological framework for assessing older adults care policy.

### 3.1 Dataset collection

This study employs yearly panel data from 2018 to 2025 to investigate the effect of policies for older adults care on quality-of-life inequality and sociodemographic risk factors among the aging population. Data by country-year were collected using three different databases that are publicly accessible, including (i) the World Bank World Development Indicators (WDI; https://databank.worldbank.org/source/world-development-indicators), providing the indicators of socioeconomics, demographics, health expenditure, and life expectancy; (ii) the World Bank Policy Database and the corresponding ASPIRE (Atlas of Social Protection Indicators of Resilience and Equity) database,^1^ providing the records of policies and social protection indicators; and (iii) the World Health Organization Global Health Observatory,^2^ providing the index of universal health coverage services and the healthy life expectancy component of the Quality of Life Index. At first, data for all countries with full information on their policy were gathered. Then, the countries with missing values for any outcomes or independent variables in any year, countries without reliable information on their policy, and countries with inconsistent information on their older adults welfare indicators (e.g., different numbers of pension coverage in WDI and ASPIRE) were excluded. This led to a balanced panel dataset of 24 countries observed over 8 years (*N* = 192 country-year observations), among which 11 belong to the treatment group and 13 to the control group (Section 3.1.1). All datasets used are publicly available, and the balanced panel, along with the estimation code, is available on request from the corresponding author (see the data availability statement).

#### 3.1.1 Definition of the policy intervention and treatment assignment

The policy intervention analyzed in this article is the adoption of an integrated older adults care policy, where an integrated older adults care policy is a nationally legislated program or strategy that (a) is specifically targeted at people aged 60 years and above; (b) includes at least two of the following four components: publicly-financed long-term care or community-based care services, broadening the health service coverage of older adults, increasing coverage of old-age social protection (pension/social assistance) and reducing out-of-pocket health costs of older adults; (c) is conducted by a national agency with a budget item for the program and (d) has an observed adoption date in the World Bank Policy Database or the corresponding national legislation. Announced policies, pilot policies that were tested subnationally, and policies covering just one component (such as an indexation adjustment for pensions) are excluded from the analysis. Table A1 in the Appendix lists the policy criteria and adoption timeline.

The assignment of treatment is done according to the aforementioned definition. In the treatment group (POLICY = 1), we have 11 countries that have adopted a qualifying policy within the sample period (2 countries in 2021 and 9 countries in 2022); in the control group (POLICY = 0), 13 countries did not have a qualifying policy active during the entire period 2018–2025. The post-treatment indicator POST takes the value of 1 starting in the year of adoption and afterward for the treated countries; for the control countries, POST is defined relative to the most common year of adoption (2022) in order to ensure that pre- and post-periods coincide in each group. Since treatment adoption occurs overwhelmingly in just one year (2022) and two earlier treatment adopters exist, our two-way fixed effects DID estimator is not affected by negative weight issues associated with staggered adoption (16, 17); lagged specification (Section 4.5.2) and event-study analysis (Section 4.3.2) help address the timing issue.

### 3.2 Variable explanation

The research makes use of various types of dependent, independent, and control variables in assessing the effects of older adults care policies, social and economic inequality, and demographic risk factors on older adults life quality and well-being. Table 1 provides a description of the variables.

This specification will allow the researcher to look at the direct impact effects (H1–H7b), as well as the effects of older adult care policies on the QoL index and LE60 under the DID assumptions. This specification will help explain how the mechanism by which socio-economic status, healthcare, demographic structure, and policy implementation affect the well-being of older adults works.

#### 3.2.1 Construction of the quality of life index

As there is no single indicator that measures well-being across all dimensions, the composite dependent variable QoL is constructed by the authors based on six indicators that form three domains of equal weight. Specifically, the health domain includes the measure of healthy life expectancy at age 60 (WHO Global Health Observatory) and the probability of death in the age interval between 30 and 70 years due to non-communicable diseases (WDI; reverse coded). The economic security domain includes the percentage of people who are past the statutory retirement age and who receive an old-age pension (ASPIRE) and the poverty rate among those aged 60+ (ASPIRE; reverse coded). The social participation domain includes the percentage of people aged 60+ who use the Internet (WDI / International Telecommunication Union, ITU) and the percentage of people aged 60+ who believe that they can rely on their relatives and friends when they need help (WDI; Gallup World Poll social support indicator). Each indicator is normalized to the [0,100] scale over the pooled sample; negative indicators are reverse-coded such that the higher the value, the better the outcome; domain scores are simply the arithmetic averages of their indicators; and the index score is simply the arithmetic average of the three domain scores, as per Equation 1:

$$\mathrm{Qo}L_{\mathrm{it}}=\frac{1}{3}\sum_{d=1}^{3}\frac{1}{K_{d}}\sum_{k=1}^{K_{d}}\frac{x_{\mathrm{kit}}-x_{k}^{\min}}{x_{k}^{\max}-x_{k}^{\min}}\times 100$$ (1)

where $x_{\mathrm{kit}}$ is the value of the indicator k for country i in year t , $K_{d}$ is the number of indicators in the domain d , and $x_{k}^{\min}$ and $x_{k}^{\max}$ are the pooled-sample minimum and maximum of the indicator k . Equal weights in the domains were selected since no theory justifies assigning more importance to any domain, and equal weights have been the default option for creating composite indices such as the Human Development Index. The index shows adequate internal consistency (Cronbach’s α = 0.82 based on six normalized indicators) and the results of the principal component analysis (PCA) performed on six indicators reveal a prominent first component accounting for 61% of variance which correlates with the equal weight index at r = 0.96; the DID estimator based on the PCA index differs insignificantly from the DID estimator calculated for the index with equal weights (3.79 versus 3.84 points). Convergent validity is confirmed by the correlation coefficients of r = 0.58 with LE60 and r = 0.52 with GDP per capita. Indicators included in the index are different from the regressors and controls presented in Table 1; hence, the index is free from mechanical overlaps with UHC, SPC, OOP, and EEMP.

### 3.3 Statistical tests

The method that is used to obtain a strong and consistent estimation of the impact of the policies on the old age care and differences in quality of life, as well as sociodemographic risk factors, is the application of a combination of descriptive and panel econometric methods. The distribution and features of the variables are analyzed using the calculation of the mean, standard deviation, minimum, and maximum values of the old-age welfare, healthcare accessibility, demographic burden, and socioeconomic environment of the sample countries using the descriptive statistics method.

The impact of the older adult care policy is estimated by the DID model by comparing the effect between the treatment and control groups before and after implementing the policy. Fixed-effects panel regression is employed in order to control for any possible country-specific and time-specific unobservable heterogeneity influencing the well-being of older adults. Statistical tests are based on robust standard errors to deal with the issue of serial correlation that causes inflated statistics in the difference-in-differences test (18). 95% confidence intervals are reported alongside *p*-values for the key policy coefficients. To further strengthen the DID identification assumption and investigate time-varying effects of the policy, a parallel trend test and an event study have been carried out. To reduce selection bias, the PSM-DID framework is presented in this article. Robustness checks are also conducted in the article to examine the validity of the empirical results.

### 3.4 DID model specification

This research uses a systematic econometric framework to investigate the effects of old-age care policy on quality-of-life gaps and older adult welfare outcomes across countries. Both socioeconomic and demographic direct influences, as well as the effects of policy (identified within a DID framework), are measured in the model, which focuses on measuring the joint impact of access to health care, economic conditions, income inequality, and demographic dependency on welfare outcomes after controlling for country- and time-specific effects.

#### 3.4.1 Baseline DID model

The baseline DID model estimates the effect of older adult care policy implementation on the Quality-of-Life Index (QoL). The model is specified as Equation 2

$$\begin{array}{l} \mathrm{Qo}L_{\mathrm{it}}=\alpha +\beta_{1}(\text{POLIC}Y_{i}\times \mathrm{POS}T_{t})+\beta_{2}\text{GDPP}C_{\mathrm{it}}\\ +\beta_{3}\mathrm{GIN}I_{it}+\beta_{4}\mathrm{UH}C_{\mathrm{it}}+\beta_{5}\mathrm{ED}R_{\mathrm{it}}+\mu_{i}+\lambda_{t}+\varepsilon_{\mathrm{it}} \end{array}$$ (2)

where i represents the country, t represents the year, and $\mathrm{Qo}L_{\mathrm{it}}$ denotes the Quality of Life Index of the country i in year t . α is the intercept term, $\text{POLIC}Y_{i}\times \mathrm{POS}T_{t}$ is the DID estimator measuring the effect of older adult care policy implementation, $\text{GDPP}C_{\mathrm{it}}$ represents the economic development level, $\mathrm{GIN}I_{\mathrm{it}}$ measures income inequality, $\mathrm{UH}C_{\mathrm{it}}$ captures healthcare accessibility, $\mathrm{ED}R_{\mathrm{it}}$ indicates the pressure of demographic aging, $\mu_{i}$ and $\lambda_{t}$ denote country and year fixed effects, and $\varepsilon_{\mathrm{it}}$ is the error term capturing unobserved factors that affect the well-being of older adults. The control variables listed in Table 1 (POV, EDU, HEXP, OOP, SPC, and EEMP) are added in the full specification reported in Section 4.

This model enables the research to estimate the direct effects of socioeconomic conditions and the DID-identified effect of older adult care policies on older adult quality-of-life outcomes.

#### 3.4.2 Extended DID model

To further examine older adult health outcomes, an extended DID model is estimated using Life Expectancy at Age 60 (LE60) as the dependent variable. The model is expressed as Equation 3.

$$\begin{array}{l} \mathrm{LE}60_{\mathrm{it}}=\alpha +\beta_{1}(\text{POLIC}Y_{i}\times \mathrm{POS}T_{t})+\beta_{2}\text{GDPP}C_{\mathrm{it}}\\ +\beta_{3}\mathrm{UH}C_{\mathrm{it}}+\beta_{4}\mathrm{ED}R_{\mathrm{it}}+\mu_{i}+\lambda_{t}+\varepsilon_{\mathrm{it}} \end{array}$$ (3)

where $\mathrm{LE}60_{\mathrm{it}}$ represents life expectancy at age 60 for the country i in year t , with country and year fixed effects included as in Equation 2. The model evaluates whether older adult care policy interventions significantly improve longevity outcomes while controlling for economic development, healthcare accessibility, and demographic dependency factors.

This specification enables the estimation of both direct socioeconomic effects and DID-identified policy effects on older adult welfare and healthy aging outcomes across regions.

### 3.5 PSM-DID analysis

To minimize selection bias further and strengthen the credibility of the DID estimates, this research utilizes the PSM-DID method. The PSM-DID method merges the propensity score matching approach with the DID framework so as to improve the comparability of the treatment and control groups in terms of their observed characteristics prior to the introduction of the older adult care policies.

In the first stage, following Rosenbaum and Rubin (19), propensity scores are estimated based on all observed socioeconomic, demographic, and health-related variables such as per capita GDP, Gini index, total health expenditures, poverty rate, universal health coverage, and older adult dependency ratio. Then, countries that adopt older adult care policies are matched with comparable non-treated countries using one-to-one nearest-neighbor matching on the estimated propensity score (with replacement, caliper = 0.05), and the balance of covariates after matching is verified before estimation.

After the matching process, the DID estimator is applied to the matched panel dataset to estimate the effect of older adult care policies on the QoL and LE60. The PSM-DID model is specified as follows in Equation 4.

$$Y_{\mathrm{it}}=\alpha +\beta (\text{POLIC}Y_{i}\times \mathrm{POS}T_{t})+\gamma X_{\mathrm{it}}+\mu_{i}+\lambda_{t}+\varepsilon_{\mathrm{it}}$$ (4)

where $Y_{\mathrm{it}}$ represents the dependent variables QoL and LE60, $\text{POLIC}Y_{i}$ denotes the treatment group indicator, $\mathrm{POS}T_{t}$ represents the post-policy implementation period, and $\left(\text{POLIC}Y_{i}\times \mathrm{POS}T_{t}\right)$ captures the treatment effect after matching. $X_{\mathrm{it}}$ includes the control variables, while $\mu_{i}$ and $\lambda_{t}$ represent the country and year fixed effects, respectively.

The PSM-DID approach improves the credibility of the empirical results by controlling for observable heterogeneity and enhances the validity of the older adult care policy effect on older adults’ well-being.

### 3.6 Mechanism analysis

Mechanism analysis is done by identifying the ways in which older adult care policy affects the quality of life of older adults. Three mechanisms are considered in relation to the three policy instruments identified in Section 3.1.1 above, namely: universal health coverage (UHC) which is a measure of the availability of basic health service; social protection coverage (SPC) which is a measure of the welfare network available to older adults; and out-of-pocket health expenditure (OOP) which is a measure of the cost to the family of accessing health service. Mechanism analysis is performed using a two-stage mediation approach whereby, in stage one, each mechanism variable takes the place of the outcome as the dependent variable in the DID regression model (Equation 5); thus, the interaction coefficient estimates the change in the mechanism due to the policy. Stage two involves estimating the relationship between each of the mechanism variables and the outcome, conditional on the interaction term and other covariates (Table 3). The product of the two coefficients approximates the indirect effect due to each channel, while the ratio of the sum of the three indirect effects to the total DID effect is the share of the policy effect explained by the three channels.

$$\text{Mechanis}m_{\mathrm{it}}=\alpha +\beta (\mathrm{DI}D_{\mathrm{it}})+\gamma X_{\mathrm{it}}+\mu_{i}+\lambda_{t}+\varepsilon_{\mathrm{it}}$$ (5)

where $\text{Mechanis}m_{\mathrm{it}}$ represents UHC, SPC, and OOP, $\mathrm{DI}D_{\mathrm{it}}$ denotes the older adult care policy interaction term, *X*^*it*^ represents control variables, while $\mu_{i}$ and $\lambda_{t}$ indicate country and year fixed effects.

**Table 3:** Baseline DID regression results.

Variables	QoL	LE60
DID (POLICY × POST)	3.842*** (0.742)	1.126*** (0.318)
GDPPC	0.00018*** (0.00005)	0.00007** (0.00003)
GINI	−0.214** (0.097)	−0.083* (0.046)
UHC	0.356*** (0.081)	0.124*** (0.037)
EDR	−0.291*** (0.102)	−0.118** (0.051)
POV	−0.178** (0.086)	−0.061* (0.033)
EDU	0.524*** (0.119)	0.163*** (0.054)
HEXP	0.441** (0.173)	0.148** (0.069)
OOP	−0.137** (0.062)	−0.052* (0.027)
SPC	0.284*** (0.073)	0.097** (0.041)
EEMP	0.116* (0.061)	0.048* (0.024)
Constant	41.286***	12.743***
Within R^2^	0.57	0.698
Country fixed effects	Yes	
Year fixed effects	Yes	

Robust standard errors are presented in parentheses. 95% confidence intervals for the DID coefficients are 2.39–5.30 (QoL) and 0.50–1.75 (LE60). *** *p* < 0.01, ** *p* < 0.05, and * *p* < 0.10. *N* = 192 country-year observations (24 countries, 2018–2025).

The stage-one estimates, the stage-two associations, and the resulting decomposition of the policy effect are reported in Section 4.4.

### 3.7 Heterogeneity analysis

The heterogeneity analysis is used to assess whether the effect of older adult care policies varies with the economic development level of the country. Given the sample size, the analysis splits the panel at the median of GDP per capita and re-estimates the baseline DID model separately for higher-income and lower-income countries (Section 4.7).

## 4 Results and analysis

From the analysis, the results have shown significant and statistically important relations, along with policy effects based on DID, between older adult care policies, socio-economic development, health care availability, demographic problems, and the quality of life of older adults across countries. The results are interpreted against the background of the literature review, with an emphasis on the variations in the quality of life of older adults in different countries due to policy effects.

### 4.1 Descriptive statistics

In terms of healthcare accessibility, socioeconomic disparities, quality of life in old age, and demographic difficulties associated with aging, the descriptive statistics show broad trends of the dataset’s primary characteristics. These figures indicate initial perceptions of variations in the distribution of study variables and aid in initial econometrics estimations. The mean (*X̄*)represents the average value of a variable and is calculated as Equation 6

$$\bar{X}=\frac{1}{N}\sum_{i=1}^{N}X_{i}$$ (6)

where $X_{i}$ represents the value of the variable for the $i^{\mathrm{th}}$ observation, and N denotes the total number of observations. In this research, $X_{i}$ may represent QoL, LE60, GDPPC, UHC, or EDR values across countries and years.

The standard deviation (SD) measures the dispersion of each variable around the mean and is calculated as Equation 7

$$\mathrm{SD}=\sqrt{\frac{1}{N-1}\sum_{i=1}^{N}{\left(X_{i}-\bar{X}\right)}^{2}}$$ (7)

where SD represents the standard deviation measuring the variation of older adult welfare and socioeconomic indicators across countries, $X_{i}$ denotes the value of a specific variable, such as QoL, LE60, GDPPC, UHC, or EDR, for the $i^{\mathrm{th}}$ country observation, $\overset{`}{X}$ represents the mean value of the variable, N indicates the total number of observations in the panel dataset, and ${\left(X_{i}-\overset{`}{X}\right)}^{2}$ measures the squared deviation of each observation from the mean. Higher SD values indicate greater variation in older adults welfare conditions, healthcare accessibility, or demographic dependency across countries. Descriptive statistics of the quality of life, socioeconomic, health care, demographic, and other policy factors for 24 countries and 192 country-year observations are presented in Table 4.

**Table 4:** Descriptive statistics of older adults care policy and quality-of-life variables.

Type	Variable	Mean	Std. Dev.	Min	Max
Dependent	QoL	68.45	11.72	42.18	89.63
	LE60	21.84	3.15	14.72	28.36
Independent	GDPPC	18543.62	12470.35	1452.18	52136.47
	GINI	37.26	7.84	24.18	58.42
	UHC	71.38	13.27	39.65	92.84
	EDR	18.47	6.91	7.24	36.58
Treatment	POLICY	0.46	0.49	0	1
	POST	0.51	0.50	0	1
Control	POV	16.82	9.64	2.15	41.37
	EDU	9.27	2.84	3.42	14.68
	HEXP	6.84	2.15	2.47	12.63
	OOP	29.63	12.41	8.16	58.72
	SPC	54.28	18.73	16.35	91.42
	EEMP	24.15	8.52	9.84	43.76

On a scale of 0 to 100, the QoL index has a mean score of 68.45 and a standard deviation of 11.72, suggesting some variations among countries and wide differences in the well-being of older adults. For LE60, the average value is 21.84, which indicates differences in health status and the lifespan of older adults across countries. For GDPPC, it has high dispersion with the average value of 18543.62, reflecting significant differences in the level of economic development. For GINI, it has a mean value of 37.26, suggesting a moderate level of income inequality in the sample countries. For UHC, the average value is 71.38, suggesting differing levels of healthcare access across countries, and for EDR, the average value is 18.47, suggesting varying levels of pressure from population aging. The dummy variables of treatment POLICY and POST are balanced for observation groups. The dummy variable POLICY takes a value of 1 for 11 treated countries and 0 for 13 control countries (average = 0.46). The variable POST is coded as 1 in the post-policy year (2021/2022 onwards, depending on the policy implementation year) and 0 otherwise (average = 0.51), such that about equal proportions of data are observed in both periods. The control variables (poverty level, education, health spending, out-of-pocket payment, social security coverage, and labor participation of older adults) are highly informative and vary sufficiently for additional regression analysis. Figure 3 shows the distribution of dependent (QoL, LE60) and independent (GDPPC, GINI, UHC, EDR) variables, which confirms their adequate variability and distribution for empirical analysis.

**Figure 3:**
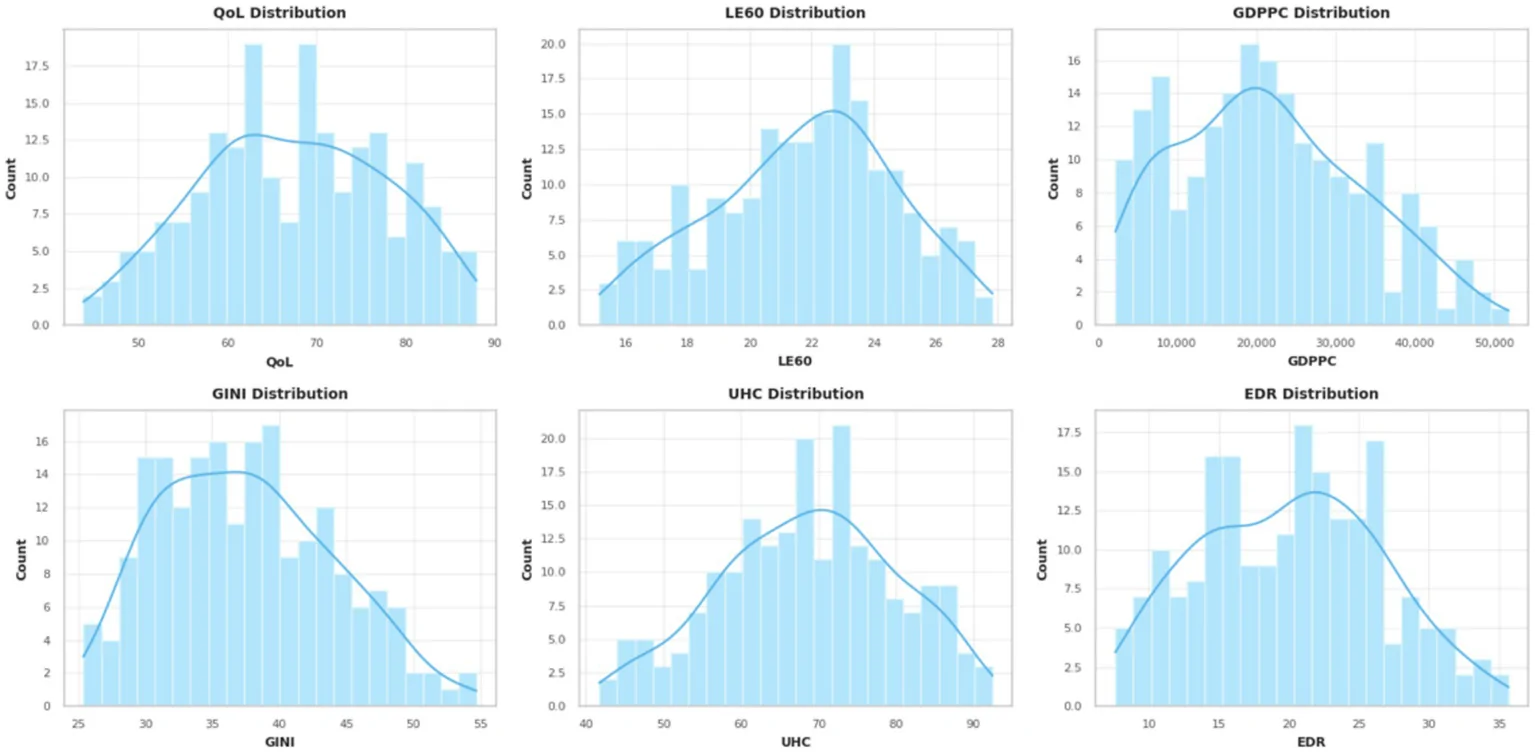
Descriptive statistics and distribution of dependent and independent variables in older adults welfare analysis.

Figure 3 indicates that both dependent and independent variables are unimodal with sufficient dispersion, without any outliers. Descriptive information, however, does not inform us about the impact of any policies; the policy effect is estimated in Sections 4.3 to 4.7.

### 4.2 Balance test

The balance test determines whether the control and treatment groups differ statistically before implementing the older adults care policy. To ensure the DID framework’s comparability assumption, the balance test is crucial. The balancing test analyses the pre-policy mean differences in pertinent health, demographic, and socioeconomic factors. The estimation using the DID technique is valid since the lack of a statistically significant difference suggests that the treatment and control groups had similar characteristics before the policy intervention. Table 5 presents the pre-policy balance test results for the treatment and control groups.

**Table 5:** Balance test between treatment and control groups before policy implementation.

Variable	Treatment mean	Control mean	Mean difference	t-statistic	*p*-value
QoL	67.82	67.35	0.47	0.62	0.534
LE60	21.47	21.31	0.16	0.48	0.629
GDPPC	18264.53	17985.41	279.12	0.73	0.468
GINI	36.92	37.15	−0.23	−0.41	0.681
UHC	70.84	70.27	0.57	0.76	0.449
EDR	18.11	17.94	0.17	0.39	0.697
POV	16.54	16.87	−0.33	−0.44	0.661
EDU	9.18	9.11	0.07	0.35	0.728
HEXP	6.72	6.65	0.07	0.29	0.771
OOP	29.41	29.76	−0.35	−0.51	0.612
SPC	53.96	53.42	0.54	0.67	0.504
EEMP	24.03	23.88	0.15	0.33	0.741

It indicates that there are no statistically significant differences in treatment and control groups in the pre-policy period, as all the *p*-values are larger than the threshold for significance. This confirms the comparability of treatment and control groups in the pre-policy period, which adds confidence in the usage of the DID method. Figure 4 indicates balanced covariates between treatment and control groups prior to the policy intervention.

**Figure 4:**
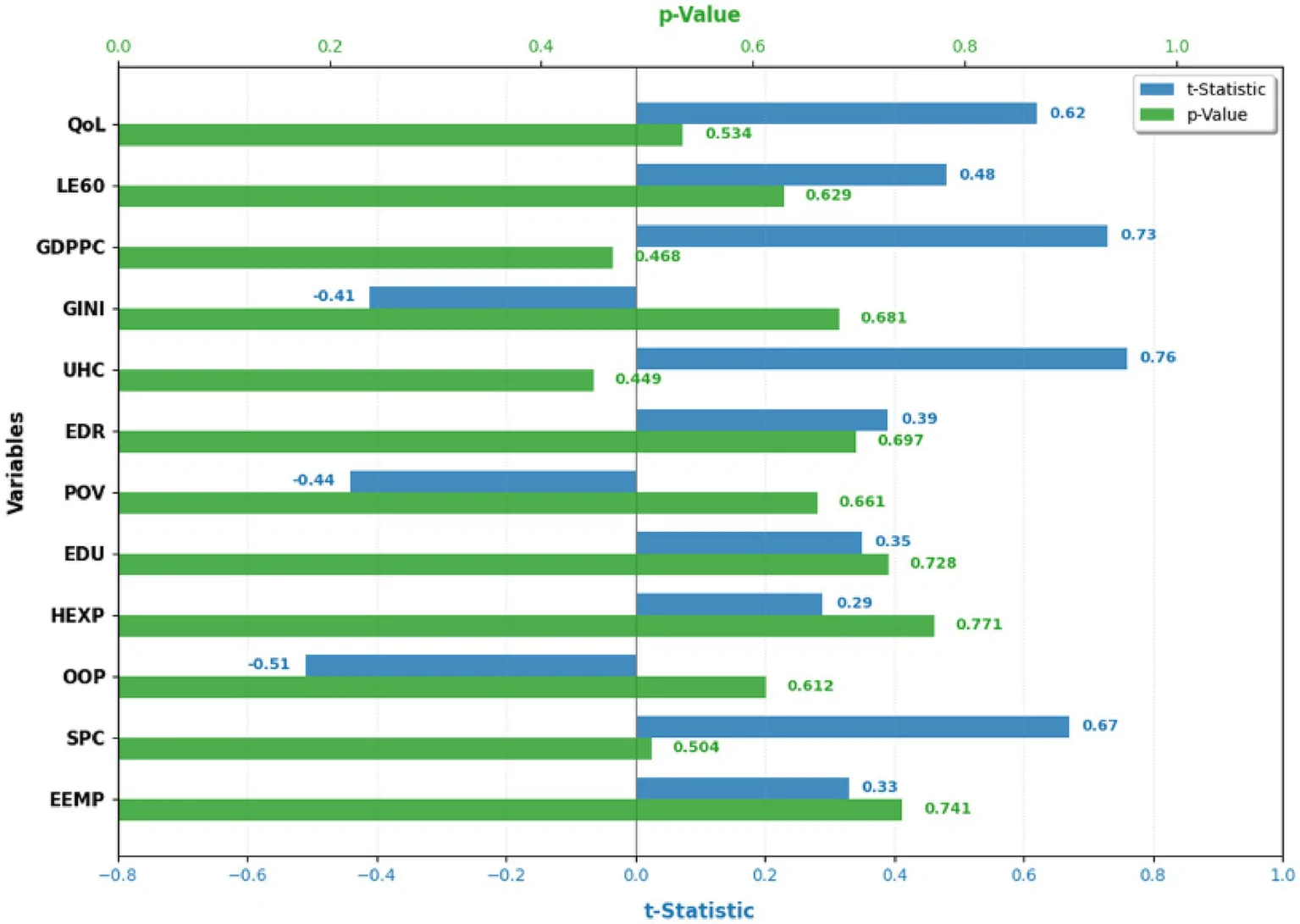
Balance test of key socioeconomic and older adults welfare indicators.

Figure 4 indicates satisfactory baseline balance in treatment vs. control groups across all major socio-economic, health, and demographic characteristics, and that there were no significant pre-policy differences between them. This lends confidence to the comparability of the two groups and strengthens the validity of the DID estimates that can be drawn using the difference-in-differences methodology.

### 4.3 Baseline DID results

The baseline DID results report the estimated effect of policy implementation on the quality of life of older adults in the treatment and control countries. The DID estimator provides the average treatment effect based on the differences in pre-post QoL and LE60 among the treatment and control countries. A regression is used to control for socio-economic, healthcare, and demographic factors. Table 3 presents the baseline DID regression results for older adult care policy impacts on QoL and LE60.

Table 3 indicates that older adult care policies are related to greater welfare (quality of life and life expectancy) for the older population in the treatment countries. The coefficients of DID are positive and significant, indicating that older adult care policies have been successful in improving the welfare of older populations after taking into consideration the influence of socioeconomic, health care, and demographic characteristics. Figure 5 provides the baseline DID estimates of the relationship between older adult care policies and QoL and LE60 outcomes.

**Figure 5:**
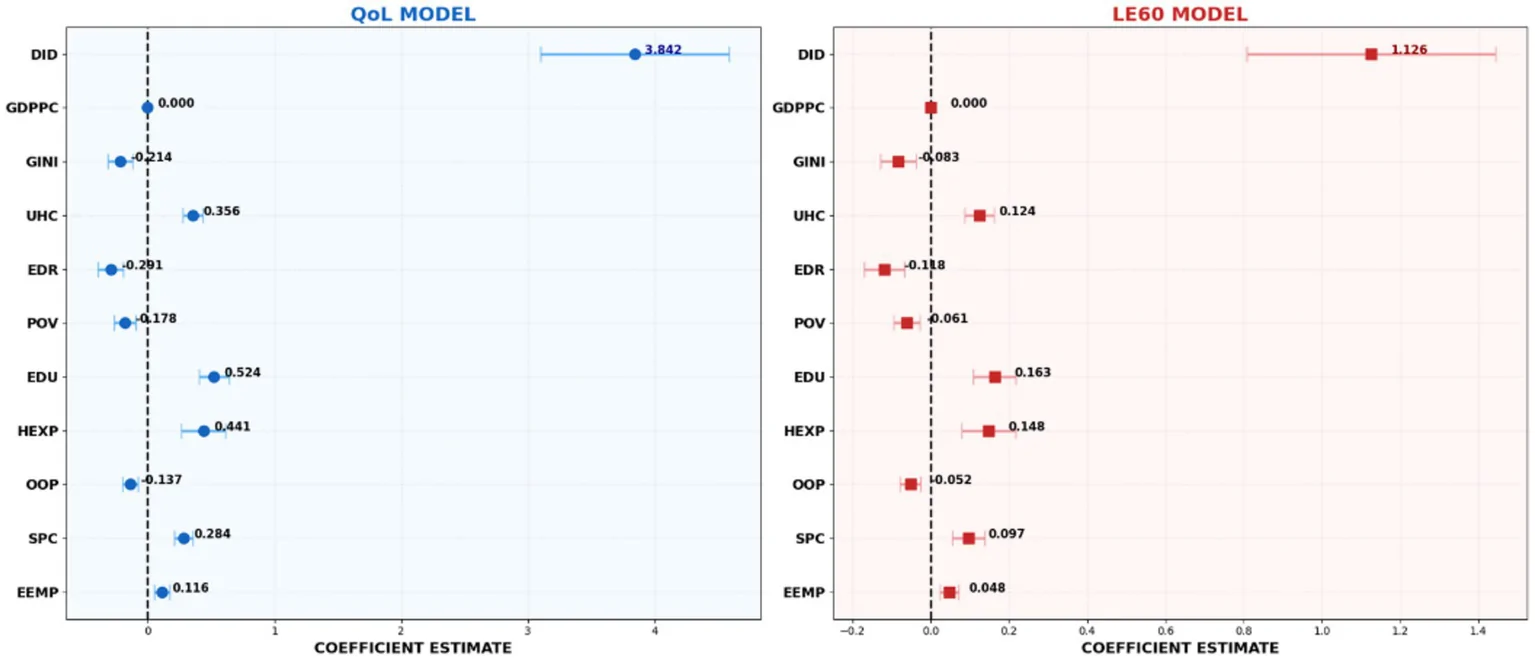
Baseline difference-in-differences (DID) estimates of older adult care policy effects on QoL and LE60.

These numbers can be interpreted as follows. The estimated QoL coefficient of 3.842 implies that, on average, in contrast with the hypothetical trajectory of QoL evolution defined by the control countries, the treated countries experienced an increase in QoL by 3.84 points on the 0–100 index after implementing the policy. It is equal to 0.33 SDs of QoL (SD = 11.72), to 5.6% of the sample mean of QoL (68.45), and to about 8% of the cross-country range (42.18–89.63). In terms of other covariates, this policy effect is comparable to the QoL change due to an increase in the UHC service index by 10.8 points (3.842/0.356) and an increase in average schooling by 7.3 years (3.842/0.524). The estimated LE60 coefficient of 1.126 implies that the life expectancy of older adults in the treated countries increased by 1.1 years on average, which is 0.36 SD (SD = 3.15) and 5.2% of the sample mean (21.84 years). During the study period, when the mean LE60 barely changed in the control countries, the gain of such magnitude is comparable to a decade of growth in longevity in the developed countries. For policy implications, these figures suggest that an integrated older adult care policy implemented at the national level generates welfare gains that are rather considerable compared to incremental changes in each socioeconomic determinant, while the confidence interval (2.39–5.30 for QoL and 0.50–1.75 for LE60) suggests some ambiguity in the effect estimation.

According to the findings from the DID, the older adult care policy has a significant effect on QoL and LE60, and education, health care expenditure, and UHC have positive effects. On the other hand, GINI and EDR have negative effects on older adult welfare in both cases.

#### 4.3.1 Parallel trends validation

According to the assumption of parallel trends, in the absence of policy, the trajectories of treated and control groups’ outcomes would be parallel. The following two arguments are put forward to support the parallel trends assumption. First, Table 6 contains the results of DID regressions using increasingly demanding specifications: the baseline interaction without covariates, the specification with covariates that corresponds to Table 3, and a full specification that additionally allows for country-specific linear time trends. The stability of the POLICY × POST coefficient across these specifications (3.218, 3.842, and 3.417), together with the pre-policy balance of QoL and of all covariates between the two groups (Table 5), indicates that the estimate is not driven by pre-existing level differences or by differential country trends. Second, and more importantly, the event study performed in section 4.3.2 provides the estimates of period-by-period treatment effects with 95% confidence intervals for three years preceding the policy period and joint testing of pre-treatment coefficients.

**Table 6:** DID estimates of older adult care policy on quality-of-life outcomes (2018–2025).

Variables	Baseline DID	DID + Controls	Full DID model
POLICY × POST (DID)	3.218*** (0.941)	3.842*** (0.742)	3.417*** (0.681)
GDPPC	-	0.00018*** (0.00005)	0.00015*** (0.00004)
GINI	-	−0.214** (0.097)	−0.186** (0.083)
UHC	-	0.356*** (0.081)	0.328*** (0.074)
EDR	-	−0.291*** (0.102)	−0.264*** (0.091)
POV	-	−0.178** (0.086)	−0.152** (0.073)
EDU	-	0.524*** (0.119)	0.483*** (0.107)
HEXP	-	0.441** (0.173)	0.397** (0.154)
OOP	-	−0.137** (0.062)	−0.121** (0.057)
SPC	-	0.284*** (0.073)	0.251*** (0.069)
EEMP	-	0.116* (0.061)	0.103* (0.052)
Country FE	Yes		
Year FE	Yes		
Within R^2^	0.41	0.57	0.63

POLICY × POST refers to the DID variable; FE stands for fixed effects. The level terms POLICY and POST are absorbed by the country and year fixed effects and are therefore not reported. The full DID model additionally includes country-specific linear time trends. Robust standard errors are presented in parentheses; the 95% confidence interval for the DID variable is 1.37–5.06 for the baseline regression, 2.39–5.30 for the regression with controls, and 2.08–4.75 for the full regression. Significance at the 10, 5, and 1% levels is indicated by *, **, and ***, respectively.

#### 4.3.2 Event study

In order to conduct the robustness check on the DID estimation, the event study approach changes the single variable of interest to a series of dummy variables that correspond to different event times before and after the implementation of the policy. Event time is specified in relation to the year in which the particular country adopts the law; the fourth year prior to the adoption year (t = −4, or 2018 for those adopting in 2022) is the omitted category. The pre-policy dummy variables (Pre-3, Pre-2, and Pre-1) evaluate whether treatment and control groups had similar paths prior to policy intervention. Similarly, post-policy dummy variables (Post-1, Post-2, Post-3) assess the impact of the dynamics of older adult care policies. The event study specification is expressed as in Equation 8.

$$\mathrm{Qo}L_{\mathrm{it}}=\alpha +\sum_{k=-3}^{+3}\beta_{k}D_{\mathrm{it}}^{k}+\gamma X_{\mathrm{it}}+\mu_{i}+\lambda_{t}+\varepsilon_{\mathrm{it}}$$ (8)

where $\mathrm{Qo}L_{\mathrm{it}}$ represents the Quality-of-Life Index for the country i at time t , $D_{\mathrm{it}}^{k}$ denotes the event-time variables for pre-policy and post-policy periods, $\beta_{k}$ captures the dynamic policy effects, $X_{\mathrm{it}}$ represents the vector of control variables, γ denotes the coefficients of control variables, $\mu_{i}$ represents country fixed effects, $\lambda_{t}$ indicates year fixed effects, and $\varepsilon_{\mathrm{it}}$ is the error term. Table 7 presents the event study estimates of older adult care policy effects on older adult quality-of-life outcomes before and after policy implementation.

**Table 7:** Event study estimates for older adult care policy effects on quality of life.

Event time variables	Coefficient	Std. error	t-statistic	*p*-value	95% CI
Pre-3	−0.218	0.412	−0.53	0.596	[−1.026, 0.590]
Pre-2	−0.147	0.368	−0.40	0.689	[−0.868, 0.574]
Pre-1	−0.083	0.291	−0.29	0.775	[−0.653, 0.487]
Current Year	1.264**	0.518	2.44	0.015	[0.249, 2.279]
Post-1	2.184***	0.637	3.43	0.001	[0.935, 3.433]
Post-2	3.027***	0.711	4.26	<0.001	[1.633, 4.421]
Post-3	3.842***	0.742	5.18	<0.001	[2.388, 5.296]
Control variables	Yes				
Country fixed effects	Yes				
Year fixed effects	Yes				
Within R^2^	0.74				

Reference period t = −4. 95% CI = 95% confidence interval derived using robust standard errors. Wald test for joint significance of the pre-policy coefficients: χ^2^(3) = 0.53, *p* = 0.91. Observations *N* = 192 country-years.

The results of the event-study analysis presented in Table 7 and Figure 6 provide clear empirical evidence regarding the trend prior to the implementation of policies. Specifically, each of the three pre-policy coefficients is relatively small in magnitude (−0.218, −0.147, and −0.083 points, that is, less than 2% of the QoL standard deviation) and individually insignificant (*p* = 0.60, 0.69, and 0.78, respectively) with 95% confidence intervals that contain zero and are narrow enough to exclude the presence of preexisting divergence greater than about one QoL point. Additionally, the joint Wald test fails to reject the null hypothesis that the three pre-policy coefficients are simultaneously equal to zero (χ^2^(3) = 0.53, *p* = 0.91). The lack of any upward trend among the pre-period coefficients also implies that the countries in the treatment group were not already experiencing an accelerated trend of improvement prior to the implementation of policies. In the post-period, the coefficients become positive and significant, growing monotonically from 1.264 (95% CI: 0.25–2.28) at the time of adoption of policies to 2.184 (95% CI: 0.94–3.43), 3.027 (95% CI: 1.63–4.42), and 3.842 (95% CI: 2.39–5.30) in the following three years.

**Figure 6:**
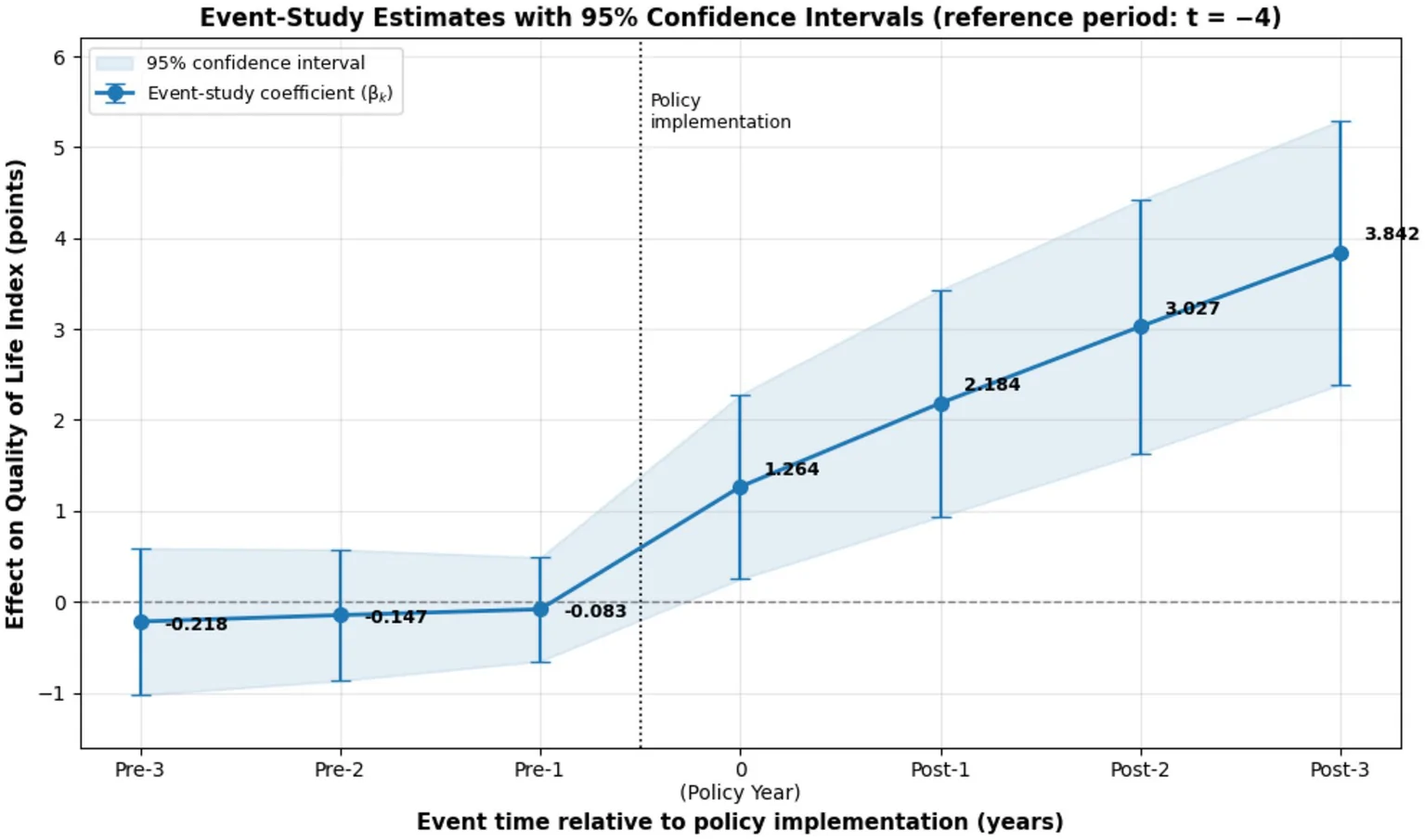
Event study estimates of older adult care policy effects on quality of life with 95% confidence intervals (reference period t = −4).

In Figure 6, we illustrate the event study coefficients and their respective 95% confidence intervals. While all of the three pre-policy confidence intervals have zero as part of the interval, this is not the case for the post-policy intervals after the adoption year, which do not include zero and increase slightly as time goes by.

### 4.4 Mechanism analysis

The mechanism analysis will help to reveal the channels that allow older adult care policies to affect quality of life through their effects on UHC, SPC, and OOP. The first-stage mechanism regressions based on Equation 5 (Table 8) show how each mechanism variable responds to the policy (DID) term.

**Table 8:** Mechanism test results of older adult care policy effects through healthcare, social protection, and financial burden pathways.

Variables	UHC	SPC	OOP
DID (POLICY × POST)	4.126*** (1.042)	5.284*** (1.317)	−3.417*** (0.984)
GDPPC	0.00014**	0.00021***	−0.00008*
GINI	−0.183*	−0.241**	0.194**
POV	−0.127*	−0.214**	0.283***
EDU	0.364***	0.417***	−0.152*
Country FE	Yes		
Year FE	Yes		
Within R^2^	0.68	0.71	0.63

Robust standard errors of the DID coefficients are presented in parentheses; *N* = 192 country-year observations. ***p < 0.01, **p < 0.05, and *p < 0.10.

Results of the mechanism test indicate that the older adult care policy increases healthcare services utilization, broadens the scope of social protection, and lowers the cost of healthcare services consumption among older adults. In particular, the policy implementation leads to an increase in the UHC service index by 4.126 points (95% CI: 2.08–6.17), an increase in social protection coverage by 5.284 points (95% CI: 2.70–7.87), and a decrease in the out-of-pocket ratio in health spending by 3.417 points (95% CI: −5.35 to −1.49). In comparison to the mean values in the sample (71.38, 54.28, and 29.63, respectively), the relative changes are 6%, 10%, and −12%, respectively.

At the second stage, connections between each of the potential mediators and the outcome variable are revealed. In accordance with the full baseline model (Table 3), the increase in the level of UHC by one point is associated with the increase in the quality of life index by 0.356 points, the increase in SPC level by one point – with the increase in the quality of life index by 0.284 points, and the increase in OOP by one point – with the decrease in the quality of life index by 0.137 points. Having combined the results from the two stages, the indirect effects of the policy via these channels were estimated at 1.47 points via the UHC channel (4.126 × 0.356), 1.50 points via the SPC channel (5.284 × 0.284), and 0.47 points via the OOP channel (−3.417 × −0.137). The total indirect effect of the policy is 3.44 points, or 90% of the total DID effect of 3.842 points. It means that the policy mainly impacts the quality of life of older adults through an increase in access to healthcare services and social protection, and a lowering of the financial burden. The logic of the mediating channels is the following: the improvement in coverage positively affects the health component of the index through detection, treatment and management of chronic diseases; better SPC coverage ensures the stability of income of older adults and reduces poverty, thus having impact on the economic security component; and reduction of out-of-pocket expenses prevents negative effects of increased health costs on other areas of people’s life. However, there are two major limitations of the decomposition approach that should be considered. First, the decomposition approach implies that the channels are independent and does not take into account the interaction of channels. Second, the second-stage coefficients show association rather than causality; thus, the shares of mediation cannot be interpreted as a proportionality measure.

### 4.5 Robustness checks

To assess the credibility and robustness of these baseline DID estimates, the analysis applies a series of alternative specifications. Robustness tests are employed to check if the obtained policy effects are driven by the use of a specific estimation technique, by the choice of variables included in the analysis, or by omitted variables and/or endogenous selection. Given the structure of the data, the following two robustness tests are performed: (1) Alternative dependent variable estimation by using LE60, and (2) Lagged policy effect estimation by using the lagged DID interaction term.

#### 4.5.1 Verifying robustness with a different dependent variable

LE60 is substituted for QoL as the dependent variable for the initial robustness test. LE60 was selected because it is a direct indicator of long-term welfare and health outcomes for older adults in relation to social security, economic stability, and health care accessibility. If older adult care policies have strong impacts on LE60, the initial results are not invalidated across alternative measures of older adult welfare. Table 9 supports the robustness of the baseline results, showing that the positive association between older adult care policies and the well-being and longevity of older adults holds for the alternative outcome.

**Table 9:** Robustness test using alternative dependent variable (LE60).

Variables	LE60
DID (POLICY × POST)	1.126*** (0.318)
GDPPC	0.00007**
GINI	−0.083*
UHC	0.124***
EDR	−0.118**
POV	−0.061*
EDU	0.163***
HEXP	0.148**
OOP	−0.052*
SPC	0.097**
EEMP	0.048*
Country FE	Yes
Year FE	Yes
Within R^2^	0.698

The findings indicate that older adult care policies show consistently positive and statistically significant effects when measuring older adult well-being through life expectancy outcomes. Robust standard errors are presented in parentheses. ***p < 0.01, **p < 0.05, and *p < 0.10.

#### 4.5.2 Robustness examination using lagged policy effects

The second robustness assessment considers a lagged DID interaction term to analyze whether the older adult care policy impacts carry over time. Lagged policy specification is meaningful since it might take time for policy outcomes concerning older adult care access, social protection schemes, and quality of life to be reflected. Lagged DID variables would catch deferred policy impacts and alleviate any potential endogeneity bias in estimation. Table 10 supports the robustness and persistence of the positive effects of older adult care policies over time.

**Table 10:** Robustness test using lagged DID variable.

Variables	QoL
Lagged DID (POLICY × POST)_t-1_	3.117*** (0.684)
GDPPC	0.00016***
GINI	−0.193**
UHC	0.337***
EDR	−0.247***
POV	−0.148*
EDU	0.462***
HEXP	0.381**
OOP	−0.116*
SPC	0.236***
EEMP	0.098*
Country FE	Yes
Year FE	Yes
Within R^2^	0.69

Standard errors are in parentheses; the 95% confidence interval for the lagged DID is 1.78–4.46. Significant at 1, 5, and 10% levels are marked by ***, **, and *, respectively.

This means that the positive impact of older adult care policies on quality-of-life variables persists even after accounting for the lagged effect of policy implementation. The robustness tests provide validation of the benchmark results obtained using DID estimation. Figure 7 supports the robustness of the baseline results, since the impact of older adult care policies remains significant and robust in different alternative and lagged models.

**Figure 7:**
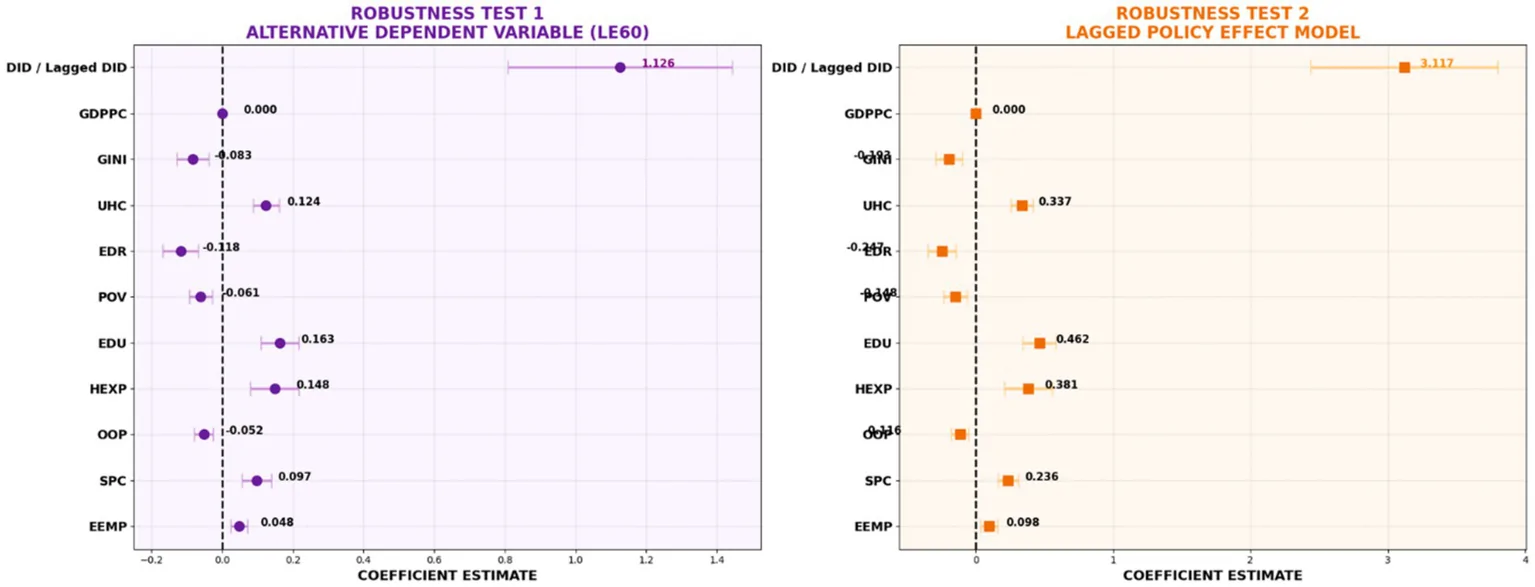
Robustness analysis of older adult care policy effects using alternative dependent variables and lagged DID specifications.

Robustness tests reveal that the effects of older adult care policies stay the same in terms of being significant and positive for older adults welfare indicators. On the other hand, control variables exhibit stable effects, whereas the levels of inequality and dependency lower older adults well-being.

### 4.6 PSM-difference-in-differences results

To minimize the potential bias resulting from selection and to increase the similarity between the treatment and control countries, PSM-DID estimation is used. The PSM procedure involves matching policy and non-policy countries on their pre-policy covariates, such as GDPPC, GINI, UHC, EDR, POV, EDU, HEXP, OOP, SPC, and EEMP, such that the matched treatment and control countries have comparable socioeconomic, healthcare, and demographic characteristics. Table 11 presents the PSM-DID estimation results for the impact of older adult care policies on quality-of-life outcomes after matching treatment and control countries.

**Table 11:** PSM-DID estimation results.

Variables	QoL	LE60
DID (POLICY × POST)	3.564*** (0.691)	1.084*** (0.301)
GDPPC	0.00016***	0.00006**
GINI	−0.191**	−0.079*
UHC	0.341***	0.119***
EDR	−0.258***	−0.111**
POV	−0.154*	−0.057*
EDU	0.472***	0.158***
HEXP	0.386**	0.141**
OOP	−0.118*	−0.048*
SPC	0.247***	0.092**
EEMP	0.101*	0.044*
Country FE	Yes	
Year FE	Yes	
Within R^2^	0.71	0.67

Parentheses are used to report robust standard errors. Significance at the 1, 5, and 10% levels is indicated by the symbols ***, **, and *.

According to PSM-DID data, the DID term’s coefficient remains positive and significant after matching. The result further indicates that older adult care policies are associated with significant improvements in the quality of life of older adults (3.564 points, 95% CI: 2.21–4.92) and in LE60 (1.084 years, 95% CI: 0.49–1.67), and that these findings are robust to selection on observables. Therefore, the positive policy effects obtained from the initial DID result are unlikely to be driven by non-comparable treatment and control countries.

### 4.7 Heterogeneity analysis by income level

To test the heterogeneous effects, the heterogeneous model is estimated for high-GDPPC and low-GDPPC groups, respectively, based on the baseline DID model. Comparing the estimated policy effects between the sub-samples could support the conclusion that economic development conditions determine policy effects and impact the narrowing of gaps in older adults well-being. Table 12 presents the heterogeneity analysis of older adult care policy effects across high- and low-income countries based on GDP per capita.

**Table 12:** Heterogeneity analysis by income level (GDP per capita).

Variables	High GDPPC	Low GDPPC
DID (POLICY × POST)	2.431** (0.984)	4.768*** (1.126)
GDPPC	0.00012**	0.00021***
GINI	−0.168*	−0.257**
UHC	0.287***	0.391***
EDR	−0.231**	−0.346***
POV	−0.137*	−0.214**
EDU	0.418***	0.572***
HEXP	0.352**	0.486**
OOP	−0.104*	−0.156**
SPC	0.223***	0.314***
EEMP	0.092*	0.134*
Country FE	Yes	
Year FE	Yes	
Within R^2^	0.69	0.75

Robust standard errors of the DID coefficients are presented in parentheses; the 95% confidence intervals of the DID coefficient are 0.50–4.36 (high-GDPPC) and 2.56–6.98 (low-GDPPC). ***p < 0.01, **p < 0.05, and *p < 0.10.

The estimation results for heterogeneity indicate that the older adult care policy exerts a significant positive impact in both high- and low-income countries. Since the DID coefficient is greater for low-income countries (4.768, 95% CI: 2.56–6.98, versus 2.431, 95% CI: 0.50–4.36), older adult care policy intervention can achieve larger welfare improvements in places with greater socio-economic disadvantage and fewer resources. Consequently, from the above analysis, it can be noted that the influence of the older adult care policy interventions on the welfare of older adults varies with the economic development level of the country, with a larger point estimate in lower-income countries. However, both confidence intervals overlap. Figure 8 shows that older adult care policy effects differ across income groups, with stronger impacts observed in low-GDPPC countries compared to high-GDPPC countries.

**Figure 8:**
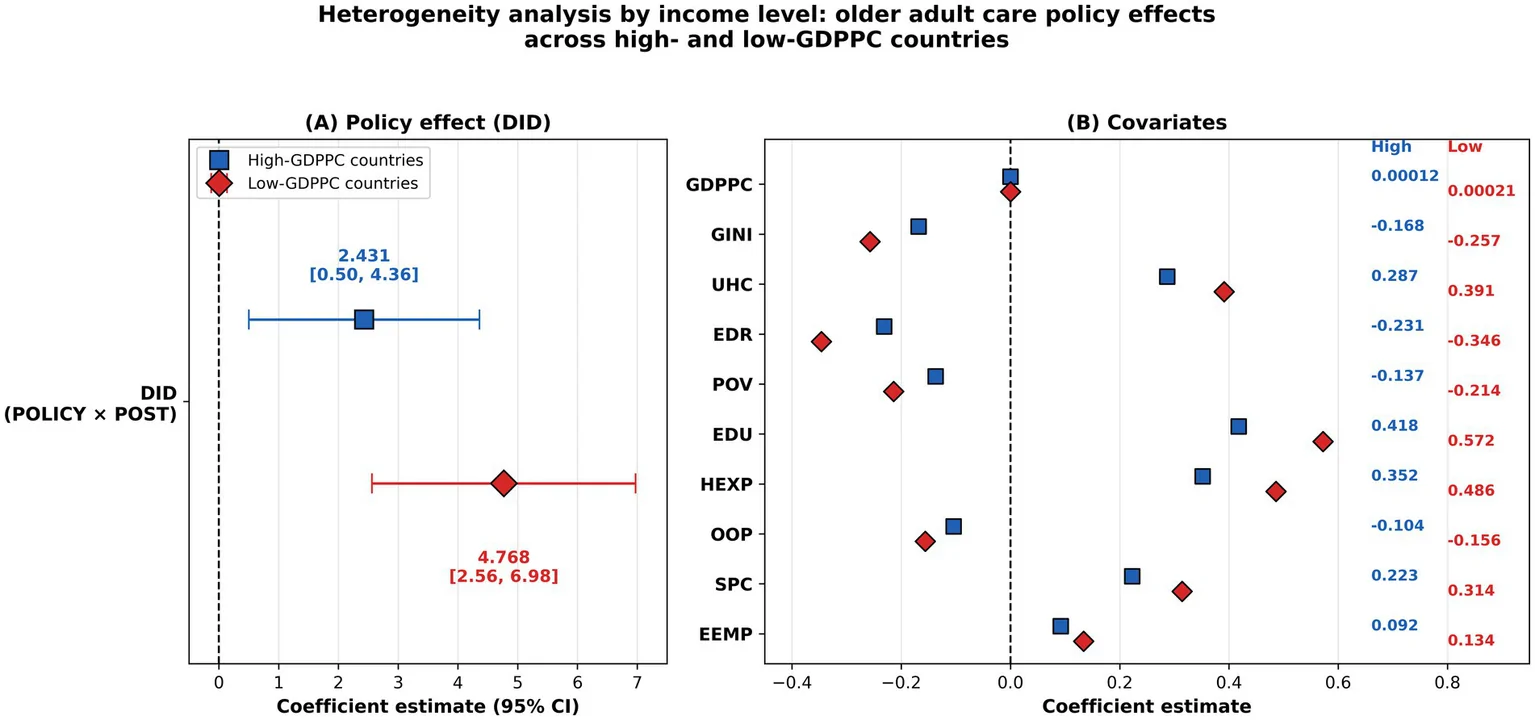
Heterogeneity analysis of older adult care policy effects across high- and low-GDPPC countries.

Heterogeneity analysis reveals that older adult care policies are more impactful in developing nations, with higher estimated gains in welfare among older adults. In addition, the impact of socioeconomic determinants is greater in these nations, indicating greater responsiveness of policies and welfare in developing economies.

## 5 Discussion

Existing research on older adults quality of life and sociodemographic differences provides limited evidence of the causal effects of older adult care policies and their capacity to mitigate disparities in quality of life by geography and demographic group. As can be observed from the literature review presented in Table 2, there are issues of causality and lack of long-term trend identification in the welfare of older adults because of the cross-sectional design; limited geographical scope which usually consists of one city or area restricts the applicability of findings to larger institutional contexts; and issues of measurement errors and reporting biases that distort observed relationships between social, health, and quality of life outcomes.

In an effort to circumvent the aforementioned shortcomings, the present study adopted the use of the longitudinal panel data approach to analyze the impact of older adult care policies on quality of life and sociodemographic disparities within aging societies. In order to strengthen identification, the study compared both the treated countries and the control group pre- and post-policy implementation using the difference-in-differences (DID) technique to find out the impact of the policies. The study relied on multi-country panel data from credible international databases and therefore was more geographically representative and applicable. Also, the inclusion of variables such as socioeconomic, demographic, health care, and welfare variables minimized the threat of omitted variable bias, while the use of techniques like fixed effects, event study analysis, robustness tests, and PSM-DID reduced the risk of unobserved heterogeneity and selection bias.

According to the findings of the DID model, the implementation of the older adult care policy was associated with significantly improved welfare of older adults. Based on the findings of the effect of the policy (POLICY × POST = 3.842, *p* < 0.01), the estimates indicate that after controlling for other socioeconomic factors and demographics, the value of QoL Index increased by about 3.84 points compared to the case where no such policies were introduced; for LE60, the positive effect of the treatment was measured at 1.126 years (*p* < 0.01). The within-R^2^ = 0.57 and 0.70 for QoL and LE60, respectively (Table 3), show that a reasonable share of variance was explained by the final model. Event-study and parallel trends analyses supported the DID framework: the coefficients Pre-3 (−0.218), Pre-2 (−0.147), and Pre-1 (−0.083) were individually and jointly insignificant (χ^2^(3) = 0.53, *p* = 0.91), consistent with the parallel trends assumption. On the other hand, the post-policy coefficients started becoming positive and statistically significant, increasing from 1.264 in the year of policy implementation to 2.184 in Post-1, 3.027 in Post-2, and 3.842 in Post-3. Results of mechanism analysis suggest that the older adult care policies have promoted life quality through different welfare mechanisms. The DID coefficients show that the policy increased UHC (4.126, *p* < 0.01) and SPC (5.284, *p* < 0.01) and reduced OOP (out-of-pocket health expenditure; −3.417, *p* < 0.01). This suggests that good health access, an adequate social protection system, and a lower financial burden are the key welfare mechanisms of older adult care policies for enhancing life quality. Regression results revealed that life quality is positively influenced by GDP per capita (GDPPC = 0.00018, *p* < 0.01), universal health coverage (UHC = 0.356, *p* < 0.01), educational level (EDU = 0.524, *p* < 0.01), health expenditure (HEXP = 0.441, *p* < 0.05), social protection coverage (SPC = 0.284, *p* < 0.01), and employment rate of older adults (EEMP = 0.116, *p* < 0.10), but negatively by income inequality (GINI = −0.214, *p* < 0.05), old-age dependency ratio (EDR = −0.291, *p* < 0.01), poverty rate (POV = −0.178, *p* < 0.05), and out-of-pocket expenditure (OOP = −0.137, *p* < 0.05). The tests of robustness, especially through PSM-DID estimators, provided the reliability of the base estimates, since the positive effect of the policies stayed statistically significant even when dealing with selection bias and model specification changes. The test of heterogeneity indicated that the older adult care policies were more effective in lower-income countries, since the DID effect was 4.768 (*p* < 0.01) in the lower-income sub-sample versus 2.431 (*p* < 0.05) in the higher-income sub-sample, although the two confidence intervals overlap.

The cross-country nature of the sample implies the existence of a potential threat, according to which differences in health systems, levels of economic development, demographic composition, and social security systems, but not the policy itself, determine the observed effect. There are a number of ways in the design of the research in order to control for the mentioned concern. First, fixed effects by country allow us to control for the time-invariant differences in countries in terms of health system type (tax-based, social insurance, or mixed), maturity of the pension system, institutions, and traditions in relation to the family obligation to take care of older adults. Second, fixed effects by year allow us to control for the shocks that affect all countries at once. This mainly concerns the coronavirus pandemic, which affected the pre-period of the research (2020–2021). Third, the time-varying control variables include the evolution of the level of economic development (GDPPC), inequality (GINI), financing of the health care system (HEXP, OOP), human capital (EDU), demographic pressure (EDR), poverty (POV), and employment of older adults (EEMP), and the balance test (Table 5) shows that the treated and the control countries do not differ statistically significantly in terms of any of these variables before adopting the policy.

Additionally, the PSM-DID methodology ensures the selection of the treated and the control countries that have similar characteristics before the adoption of the policy, while heterogeneity analysis shows that the effect exists in the subsamples of high-income and low-income countries. Therefore, the effect is not determined by the comparison of the richer and poorer countries. However, there is no way to control for the existence of potential time-varying unobserved confounders that are correlated with the adoption of the policy in the treated countries. This concerns the health system reforms, recovery from the coronavirus pandemic, and the evolution of the policy implementation quality. The demographic composition of the treated countries is also a potential unobserved confounder since the countries with a rapid increase in the dependency ratio adopt the policy on older adult care exactly in the period of increasing pressure on older adults, thus causing a downward bias in the estimates. Therefore, the conditional interpretation of the results can be the most appropriate one provided that the parallel trends condition holds after controlling for all the observable variables and fixed effects.

## 6 Conclusion

With the rapid population aging and escalating pressure on the healthcare and social welfare system, older adults welfare has been the focal point of policy concerns across the globe. Using panel data and an econometric design involving DID, event-study analysis, mechanism tests, robustness tests, PSM-DID, and heterogeneity analysis, this article provides evidence on the effects of older adult care policies on the welfare outcomes of older adults in a panel of 24 countries over 2018–2025. The results indicate that older adult care policies significantly improve the quality of life and life expectancy of older adults (by 3.84 index points and 1.13 years, respectively), and decrease cross-country welfare disparities. Mechanism testing suggests expansion of universal health coverage, a stronger social security system, and a decreasing proportion of household out-of-pocket expenditure are the main transmission channels. Robustness tests and PSM-DID analysis support the stability of the estimated policy impacts. Additionally, the heterogeneity analysis suggests that economically disadvantaged countries benefit more from the policy. Integrated policies for older adults combining access to health services, social security provision, poverty alleviation, and human capital improvement are crucial to healthy aging, improved quality of life, and equitable welfare for older adults regardless of socioeconomic status.

### 6.1 Limitations and future scope

However, several potential issues need to be considered. First, since the analysis is based on cross-country secondary data, access to the data, measurement standards, and implementation of policies are likely to differ across countries; in turn, the treatment variable reflects the presence of any qualifying national policy but not its intensity or implementation process; therefore, the results show an average effect of different policies. Second, the panel consists of 24 countries and has a maximum four-year post-policy period, which limits the statistical power and makes it impossible to conclude long-term effects. Third, while the fixed effects, time-varying controls, matching, and evidence from event studies make it less likely to have confounding variables and problems with the identification of the effect due to the parallel trends assumption, there is still no way to exclude time-varying unobservables that are correlated with the adoption of policies and the untestability of the parallel trends assumption for the post period. Finally, the mechanism decomposition depends on conditional correlations and additive channels.

## Data Availability

Publicly available datasets were analyzed in this study. These data can be found in the World Bank World Development Indicators (https://databank.worldbank.org/source/world-development-indicators), the World Bank ASPIRE database (https://www.worldbank.org/aspire), and the WHO Global Health Observatory (https://www.who.int/data/gho). The constructed country-year panel and the estimation code are available from the corresponding author upon reasonable request.

## Footnotes

1
https://www.worldbank.org/aspire

2
https://www.who.int/data/gho

## Appendix

**Table A1:** Definition of the older adult care policy intervention and treatment assignment.

Element	Specification
Policy definition	Nationally legislated integrated older adult care policy targeting persons aged 60+ that combines at least two of: publicly financed long-term/community-based care; expanded health service coverage for older adults; expanded old-age social protection coverage; measures reducing out-of-pocket health spending of older adults
Exclusions	Sub-national pilots; announced but unimplemented strategies; single-instrument adjustments (e.g., pension indexation only)
Data sources for classification	World Bank Policy Database; national legislation and program documents; ASPIRE
Treatment group (POLICY = 1)	11 countries: 2 adopted in 2021, 9 adopted in 2022
Control group (POLICY = 0)	13 countries with no qualifying policy in force during 2018–2025
POST definition	1 from the adoption year onward for treated countries; 1 from 2022 onward for control countries (modal adoption year)
Event-time window	Pre-3 to Post-3 relative to adoption; reference period t = −4; later periods binned into Post-3
Observations	24 countries × 8 years = 192 country-year observations (balanced panel)

The classification was applied uniformly to all sample countries using the sources listed above; the country-level classification file is available from the corresponding author on request.

## References

[1] Geigl C Loss J Leitzmann M Janssen C. Social factors of health-related quality of life in older adults: a multivariable analysis. Qual Life Res. (2023) 32:3257–68. doi: 10.1007/s11136-023-03472-4, 37458960 PMC10522508

[2] Malicka B Skośkiewicz-Malinowska K Kaczmarek U. The impact of socioeconomic status, general health, and oral health on health-related quality of life, oral health-related quality of life, and mental health among polish older adults. BMC Geriatr. (2022) 22:2. doi: 10.1186/s12877-021-02716-7, 34979959 PMC8722217

[3] Siqeca F Yip O Mendieta MJ Schwenkglenks M Zeller A De Geest S . Factors associated with health-related quality of life among home-dwelling older adults aged 75 or older in Switzerland: a cross-sectional study. Health Qual Life Outcomes. (2022) 20:166. doi: 10.1186/s12955-022-02080-z, 36544173 PMC9773624

[4] Santhalingam S Sivagurunathan S Prathapan S Kanagasabai S Kamalarupan L. The effect of socioeconomic factors on the quality of life of the elderly in the Jaffna district of Sri Lanka. PLoS Glob Public Health. (2022) 2:e0000916. doi: 10.1371/journal.pgph.0000916 PMC10021859 36962835

[5] Rahman MM Sorkar MNU Nakamura R Islam MM Alam MA Azmat SK . Socio-demographic disparities in health-related quality of life in hypertensive patients in Bangladesh: a comprehensive survey analysis. Qual Life Res. (2025) 34:1457–71. doi: 10.1007/s11136-025-03912-3, 39913058 PMC12064452

[6] Ribot-Rodriguez R Higuera-Gomez A San-Cristobal R Martín-Hernández R Micó V Espinosa-Salinas I . Cardiometabolic health status, ethnicity, and health-related quality of life (HRQoL) disparities in an adult population: NutrIMDEA observational web-based study. Int J Environ Res Public Health. (2022) 19:2948. doi: 10.3390/ijerph19052948, 35270641 PMC8910247

[7] Beltz S Gloystein S Litschko T Laag S van den Berg N. Multivariate analysis of independent determinants of ADL/IADL and quality of life in the elderly. BMC Geriatr. (2022) 22:894. doi: 10.1186/s12877-022-03621-3, 36418975 PMC9682836

[8] Severino R Espinoza M Cabieses B. Health-related quality of life by household income in Chile: a concentration index decomposition analysis. Int J Equity Health. (2022) 21:176. doi: 10.1186/s12939-022-01770-w, 36514033 PMC9749309

[9] Lopez J Perez-Rojo G Noriega C Sánchez-Cabaco A Sitges E Bonete B. Quality of life in older adults: its association with emotional distress and psychological well-being. BMC Geriatr. (2024) 24:815. doi: 10.1186/s12877-024-05401-7, 39385087 PMC11465940

[10] Fahmy VF Momen MAMT Mostafa NS Elawady MY. Prevalence, risk factors, and quality of life impact of work-related musculoskeletal disorders among school teachers in Cairo, Egypt. BMC Public Health. (2022) 22:2257. doi: 10.1186/s12889-022-14712-6, 36463148 PMC9719209

[11] Goyal AK Mohanty SK. Association of pain and quality of life among middle-aged and older adults of India. BMC Geriatr. (2022) 22:939. doi: 10.1186/s12877-022-03480-y, 36474187 PMC9724285

[12] Agyemang-Duah W Abdullah A Rosenberg MW. Caregiver burden and health-related quality of life: a study of informal caregivers of older adults in Ghana. J Health Popul Nutr. (2024) 43:31. doi: 10.1186/s41043-024-00509-3, 38383532 PMC10882722

[13] Shin J Shin DW Lee J Hwang J Lee JE Cho B . Exploring socio-demographic, physical, psychological, and quality of life-related factors related to fear of cancer recurrence in stomach cancer survivors: a cross-sectional study. BMC Cancer. (2022) 22:414. doi: 10.1186/s12885-022-09507-2 PMC9013039 35428224

[14] Hagan K Javed Z Cainzos-Achirica M Hyder AA Mossialos E Yahya T . Cumulative social disadvantage and health-related quality of life: National Health Interview Survey 2013–2017. BMC Public Health. (2023) 23:1710. doi: 10.1186/s12889-023-16168-8, 37667245 PMC10476290

[15] Chang TG Huang PC Hsu CY Yen TT. Demoralization in oral cancer inpatients and its association with spiritual needs, quality of life, and suicidal ideation: a cross-sectional study. Health Qual Life Outcomes. (2022) 20:60. doi: 10.1186/s12955-022-01962-6, 35366908 PMC8976948

[16] Goodman-Bacon A. Difference-in-differences with variation in treatment timing. J Econ. (2021) 225:254–77. doi: 10.1016/j.jeconom.2021.03.014

[17] Callaway B Sant'Anna PHC. Difference-in-differences with multiple time periods. J Econ. (2021) 225:200–30. doi: 10.1016/j.jeconom.2020.12.001

[18] Bertrand M Duflo E Mullainathan S. How much should we trust differences-in-differences estimates? Q J Econ. (2004) 119:249–75. doi: 10.1162/003355304772839588

[19] Rosenbaum PR Rubin DB. The central role of the propensity score in observational studies for causal effects. Biometrika. (1983) 70:41–55. doi: 10.1093/biomet/70.1.41

[20] Rico-Blázquez M Quesada-Cubo V Polentinos-Castro E Sánchez-Ruano R Rayo-Gómez MÁ del Cura-González I. Health-related quality of life in caregivers of community-dwelling individuals with disabilities or chronic conditions. A gender-differentiated analysis in a cross-sectional study. BMC Nurs. (2022) 21:69. doi: 10.1186/s12912-022-00845-x, 35351100 PMC8961096

[21] Yao Q Zhang X Wu Y Liu C. Decomposing income-related inequality in health-related quality of life in mainland China: a national cross-sectional study. BMJ Glob Health. (2023) 8:e013350. doi: 10.1136/bmjgh-2023-013350, 38035731 PMC10689391

[22] Ghanem AS Móré M Nagy AC. Assessing the impact of sociodemographic and lifestyle factors on oral health: a cross-sectional study in the Hungarian population. Front Public Health. (2023) 11:1276758. doi: 10.3389/fpubh.2023.1276758, 37927886 PMC10623121

[23] Alliu I Thapa S Yu L Shehaj B Asifat O. Economic and humanistic burden of multimorbidity in the United States: a longitudinal study of expenditure and quality of life trajectories, 2019–2022. Int J Environ Res Public Health. (2025) 22:1870. doi: 10.3390/ijerph22121870, 41464503 PMC12733161

[24] Liu S Lu Y Wang D He X Ren W Kong D . Impact of digital health literacy on health-related quality of life in Chinese community-dwelling older adults: the mediating effect of health-promoting lifestyle. Front Public Health. (2023) 11:1200722. doi: 10.3389/fpubh.2023.1200722, 37415711 PMC10321557

[25] Sieber S Roquet A Lampraki C Jopp DS. Multimorbidity and quality of life: the mediating role of ADL, IADL, loneliness, and depressive symptoms. Innov Aging. (2023) 7:igad047. doi: 10.1093/geroni/igad047, 37435089 PMC10332504

[26] Ramalivhana FW Veldsman T Moss SJ. Assessment of non-communicable disease risk factors, functional performance, and health-related quality of life in adults: a comparative analysis in low-resourced urban and rural areas of South Africa. BMC Public Health. (2024) 24:1580. doi: 10.1186/s12889-024-18964-2, 38867182 PMC11170915

[27] Prati G. Correlates of quality of life, happiness, and life satisfaction among European adults older than 50 years: a machine-learning approach. Arch Gerontol Geriatr. (2022) 103:104791. doi: 10.1016/j.archger.2022.104791 35998473

[28] Nagy E Tharwat S Elsayed AM Shabaka SAEG Nassar MK. Anxiety and depression in maintenance hemodialysis patients: prevalence and their effects on health-related quality of life. Int Urol Nephrol. (2023) 55:2905–14. doi: 10.1007/s11255-023-03556-7, 37009953 PMC10560136

[29] Geprägs A Bürgin D Fegert JM Brähler E Clemens V. The impact of mental health and sociodemographic characteristics on quality of life and life satisfaction during the second year of the COVID-19 pandemic—results of a population-based survey in Germany. Int J Environ Res Public Health. (2022) 19:8734. doi: 10.3390/ijerph19148734, 35886588 PMC9316196

[30] Muneera K Muhammad T Althaf S. Socio-demographic and lifestyle factors associated with intrinsic capacity among older adults: evidence from India. BMC Geriatr. (2022) 22:851. doi: 10.1186/s12877-022-03558-7, 36368936 PMC9652958

[31] Mihevc M Potočnik TV Zavrnik Č Šter MP Klemenc-Ketiš Z Susič AP. Beyond diagnosis: investigating factors influencing health-related quality of life in older people with type 2 diabetes in Slovenia. Prim Care Diabetes. (2024) 18:157–62. doi: 10.1016/j.pcd.2024.01.010, 38320938

[32] Liang X Wei H Mo H Yang G Wan L Dong H . Impacts of chronic diseases and multimorbidity on health-related quality of life among community-dwelling elderly individuals in economically developed China: evidence from a cross-sectional survey across three urban centers. Health Qual Life Outcomes. (2024) 22:91. doi: 10.1186/s12955-024-02309-z, 39462427 PMC11515108

[33] Ko Y Lee K. Social frailty and health-related quality of life in community-dwelling older adults. Int J Environ Res Public Health. (2022) 19:5659. doi: 10.3390/ijerph19095659, 35565056 PMC9100126

[34] Bally EL Korenhof SA Ye L van Grieken A Tan SS Mattace-Raso F . Factors associated with health-related quality of life among community-dwelling older adults: the APPCARE study. Sci Rep. (2024) 14:14351. doi: 10.1038/s41598-024-64539-x PMC11192871 38906882

[35] Wang D Xie S Wu J Sun B. The trend in quality of life of the Chinese population: analysis based on population health surveys from 2008 to 2020. BMC Public Health. (2023) 23:167. doi: 10.1186/s12889-023-15075-2 PMC9873389 36694154

